# ColonyArea: An ImageJ Plugin to Automatically Quantify Colony Formation in Clonogenic Assays

**DOI:** 10.1371/journal.pone.0092444

**Published:** 2014-03-19

**Authors:** Camilo Guzmán, Manish Bagga, Amanpreet Kaur, Jukka Westermarck, Daniel Abankwa

**Affiliations:** 1 Turku Centre for Biotechnology, University of Turku and Åbo Akademi University, Turku, Finland; 2 Department of Pathology, University of Turku, Turku, Finland; 3 Turku Doctoral Program of Biomedical Sciences, University of Turku and Åbo Akademi University, Turku, Finland; Ospedale Pediatrico Bambino Gesu', Italy

## Abstract

The clonogenic or colony formation assay is a widely used method to study the number and size of cancer cell colonies that remain after irradiation or cytotoxic agent administration and serves as a measure for the anti-proliferative effect of these treatments. Alternatively, this assay is used to quantitate the transforming potential of cancer associated genes and chemical agents. Therefore, there is a need for a simplified and standardized analysis of colony formation assays for both routine laboratory use and for parallelized automated analysis. Here we describe the freely available ImageJ-plugin “ColonyArea”, which is optimized for rapid and quantitative analysis of focus formation assays conducted in 6- to 24-well dishes. ColonyArea processes image data of multi-well dishes, by separating, concentrically cropping and background correcting well images individually, before colony formation is quantitated. Instead of counting the number of colonies, ColonyArea determines the percentage of area covered by crystal violet stained cell colonies, also taking the intensity of the staining and therefore cell density into account. We demonstrate that these parameters alone or in combination allow for robust quantification of IC_50_ values of the cytotoxic effect of two staurosporines, UCN-01 and staurosporine (STS) on human glioblastoma cells (T98G). The relation between the potencies of the two compounds compared very well with that obtained from an absorbance based method to quantify colony growth and to published data. The ColonyArea ImageJ plugin provides a simple and efficient analysis routine to quantitate assay data of one of the most commonly used cellular assays. The bundle is freely available for download as supporting information. We expect that ColonyArea will be of broad utility for cancer biologists, as well as clinical radiation scientists.

## Introduction

Since the introduction of clonogenic assays in 1956 by Puck and Marcus [1], they have become the method of choice to determine the survival and growth of cells, in particular cancer cell lines, after treatment with ionizing radiation or to determine the effectiveness of cytotoxic agents [2]–[4]. A clonogenic assay evaluates the potential of a single cell to resist treatments and grow into a colony, which lent the assay the alternative name of colony formation assay [3]. In addition, the colony formation assay has also gained significance to evaluate the transforming or colony growth potential of oncogenes, such as H-ras or CIP2A [5]–[7].

Traditionally clonogenic assays have been performed by counting colonies or foci of cells, which typically comprised >50 densely-packed cells [1], [3]. Cells are usually identified by staining with a crystal violet dye [3], which primarily binds to polyanionic sugar molecules such as DNA in the nucleus of mammalian cells [8]. If solubilized from stained cells, measuring the absorption of the crystal violet dye can be used to quantify cellular growth [9], however with the disadvantage that the cellular sample is destroyed.

Colony counting can be done either with the slow and subjective, manual (human) counting or using a large variety of devices and programs that accelerate and automate counting [2], [4], [10]–[13]. The major image analysis challenges are the identification and separation of colonies, as well as integration of the sizes of colonies. Colony number and size would reflect cell survival and proliferation, respectively. Image analysis may require expensive equipment (e.g. GelCount hardware described in [10]), or the use of commercialized software (ScanCount, [2] and MetaMorph, [11]). Alternatively, free distribution software can be employed, either as standalone solutions (CellProfiler, [12] and OpenCFU, [13]) or as macros for ImageJ (National Institutes of Health, Bethesda MD – USA) [4].

As an alternative to counting and quantifying individual colonies, it is much simpler to determine the percentage of the well area that is covered by colonies (colony area percentage) to quantify clonogenic cell growth [7], [14]–[17]. We here describe the ImageJ plugin, “ColonyArea”, which determines the colony area percentage and an intensity weighted colony area percentage (colony intensity percentage) from flatbed scanner acquired images of colony formation assays conducted in multi-well plates. The plugin is user-friendly, as it basically only requires 1) the selection of a rectangular ROI (region of interest) that encompasses wells to be analyzed, and 2) choice of the well-plate type by the user. We test our plugin by quantifying the susceptibility of T98G human glioblastoma cell growth to two different staurosporines, UCN-01 and staurosporine (STS), both well known inhibitors of protein kinases and prominent anti-proliferative drugs [18]–[21]. We validate the accuracy of our results by recovering the different potencies of UCN-01 and STS, as well as by direct comparison of obtained data with data generated by the absorption based method from Kueng *et al.* [9].

## Results and Discussion

**Figure 1:**
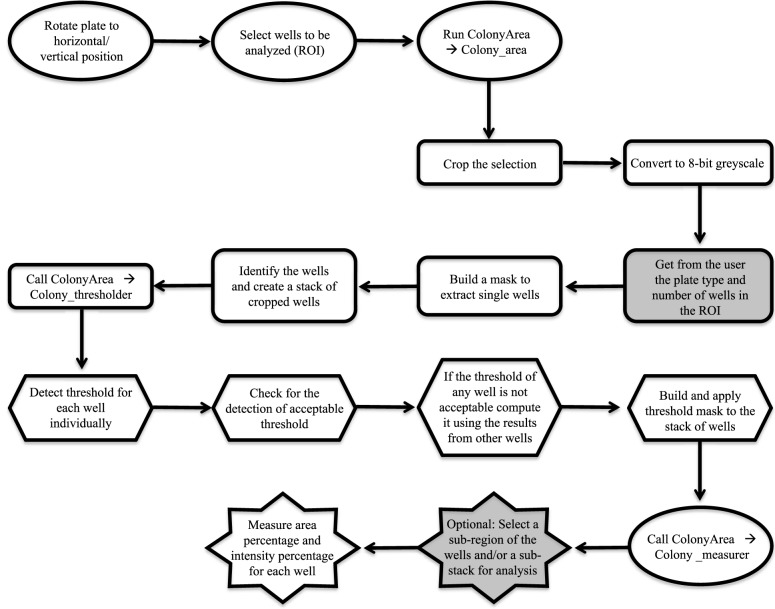
Flow chart of the processing steps in the ColonyArea plugin. Steps performed by the user are represented by ovals and the grey shapes are those requiring user input. All other shapes represent steps performed by the three macros Colony_area (rounded rectangles), Colony_thresholder (hexagons) and Colony_measurer (stars) that are packaged as one plugin file.

We have developed “ColonyArea”, a java-based free distribution plugin for the open-source image analysis software ImageJ. ColonyArea precisely and rapidly quantifies scanned images of colony formation assays (**Figure 1**). It is already set up to operate on standard 6-, 12- and 24-well cell culture plates, and can be further customized to handle other multi-well formats. In the following, we describe how the plugin automatically separates all user-selected wells in an image, eliminates the background and quantifies colony formation.

An additional file placed as **Information S1** and termed ‘ColonyArea.zip’, contains the plugin and user manual. This file is also freely available on our website http://www.btk.fi/research/research-groups/abankwa/downloads/ and on the webpage of the European Data Infrastructure (EUDAT) https://b2share.eudat.eu/record/45 through their service B2SHARE.

### Automatic well separation and cropping

**Figure 2:**
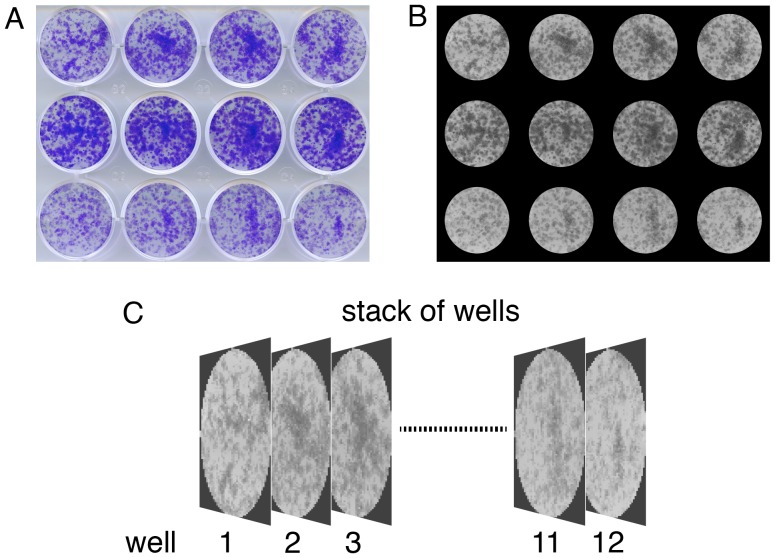
Identification of wells and generation of a well image stack. (**A**) Scanned image of a 12-well plate showing different levels of colony formation of drug treated T98G human glioblastoma cells. (**B**) Same image as in (A) after automatic identification of the wells. The image was converted into an 8-bit greyscale and spaces between wells were removed using a mask. (**C**) Each well image was then concentrically cropped and added to an image stack to allow for the analysis of each well individually.

The ColonyArea plugin consists of a bundle of the actual java-file and macros. For simplicity we refer to this bundle as the ‘plugin’. The plugin procedure uses a scanned image of a multi-well assay plate (**Figure 2A**) and after separating individual wells performs all processing and analysis steps well-specifically, for example background thresholding. In order to separate the wells from a plate image, the java file “Colony_area” starts by using information about the plate type and the number of selected wells, which are both provided by the user to create a mask that will set the intensity of those pixels belonging to the space in between wells to zero. The size and shape of the mask depend on the above user information and typical plate dimensions of 6- to 24-well plates. Dimensions as published by a major manufacturer (CELLSTAR, Greiner Bio-One) are stored in the plugin. Manual comparison with plates from Millipore and BD Biosciences gave similar dimensions. However, it is possible that the user provides these dimensions, as detailed in the manual. Next this image is converted into a grey scale 8-bit image (**Figure 2B**). To circumvent issues associated with cell growth abnormalities on the well edges, these are eliminated from analysis using a concentrical cropping mask, which reduces each well diameter by 5% from the edges. Subsequently, the macro separates and further crops each well image such that the edge of the well is aligned with the edges of the individual image. In the last step, an image stack is created, containing all the wells that were selected from the original image (**Figure 2C**).

### Identification of the background with “Colony_thresholder”

**Figure 3:**
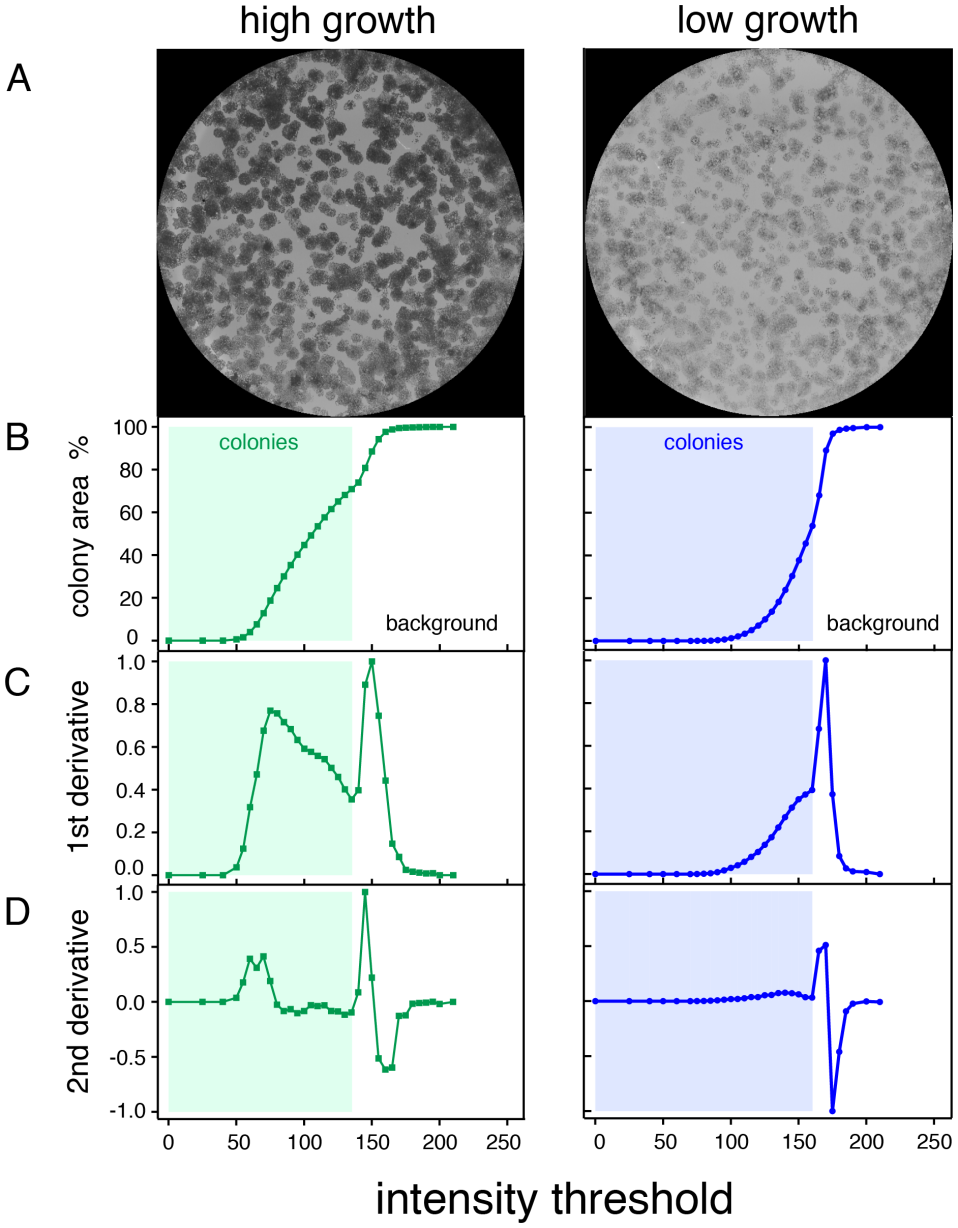
Determination of the background threshold. (**A**) Two 8-bit greyscale images of wells showing high (**left**) and lower (**right**) intensities of cell staining with similar colony density. (**B**) For each case, the colony area percentage is plotted as a function of the applied intensity threshold. At this point, the colony area percentage corresponds to the percentage of the well area that is selected based on the criterion that each pixel in the area has an intensity value below a given intensity threshold. (**C**) First and (**D**) second derivatives of the colony area percentage function shown in (**B**), which allow identifying the correct intensity threshold. After the correct threshold has been identified, the colony area parameter gives the percentage of the well area that is occupied by cells. In all plots (**B–D**), the highlighted region represents the intensity range where only cells are selected. Above that intensity threshold the background starts to be included, which identifies this intensity value as the background threshold.

Since colony formation assays typically involve staining of the cells with a crystal-violet dye [3], pixels corresponding to regions with cells will appear darker and have smaller grey-values than those without cells. The macro “Colony_thresholder” of our plugin recognizes these differences in intensity and determines per well image the intensity value that separates the background (high intensity values) from stained cell colonies (low intensity values), in other words the background threshold (**Figure 3**).

If we plot the colony area percentage function in dependence of an applied intensity threshold, i.e. for now the area selected with an intensity value below a given intensity threshold, we observe that above a certain intensity threshold value the selected area function suddenly increases to 100% (**Figure 3B**). This is the intensity value, above which also high grey-value/ intensity pixels of the background are selected wrongly as ‘cells’ and it therefore corresponds to the actual background threshold. Identification of this transition point is facilitated, by determining the first (**Figure 3C**) and second derivative (**Figure 3D**) of the area percentage function, which identifies this point as a local minimum or zero-intercept on the x-axis, respectively. The algorithm inside Colony_thresholder calculates these three functions and explores them to identify the transition point that represents the background threshold.

**Figure 4:**
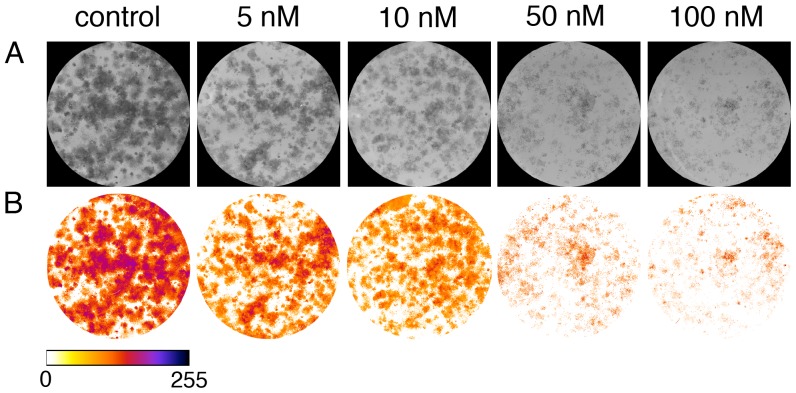
Removal of the background. (**A**) 8-bit greyscale images of individual wells showing different levels of colony formation of T98G cells treated with indicated concentrations of staurosporine. (**B**) Same individual wells after thresholding and background removal by the macro “Colony_thresholder”. Color bar represents the intensity scale displayed in the thresholded wells. Zero intensity (white) corresponds to areas where no cells were identified (background).

Since there is a possibility that there are multiple local minima and zero-intercepts on the second and third derivative, which would hamper automatic threshold identification, the obtained background thresholds from wells of the same plate are then undergoing a consistency check. First, the maximum intensity in the wells is computed and all the well images are linearly scaled such that their maximum intensity is 200. This is done to counter the effect of non-uniform illumination, or any backlight correction of the scanner. Then the scaled threshold values are compared and any value that deviates more than 50 intensity units from the average is flagged for reevaluation. In this case, the background threshold for that particular well is recalculated restricting the above background analysis procedure to intensity values within the range of the average background threshold ±50 intensity units, instead of using the full, initial range of 0 to 255. Then the threshold obtained from the restricted range is scaled back to the initial intensity values and applied to a copy of the initial well image. In a last step, ColonyArea uses these thresholded copy images and the initial 8-bit images of the wells (**Figure 4A**), to create a new set of images, where the intensity is zero for pixels that do not contain cells and a number between 1 and 255 for pixels that do contain cells. Images in this new set are then inverted so higher intensity values reflect the increasing density of cells at any given pixel (**Figure 4B**). These thresholded images are used in subsequent quantitation steps.

In order to allow the user to verify correct background identification, ColonyArea displays the stacks of well images before and after thresholding side-by-side. In case the user is not satisfied with the result, ColonyArea is equipped with an option for user defined manual thresholding, which is described in the plugin manual provided with the download bundle.

### Quantification of colony growth with “Colony_measurer”

Our plugin automatically identifies the surface area occupied by cells in a well and calculates from this the output parameter ‘colony area percentage’, i.e. the percentage of well area covered with cells. This simplified quantification for colony formation assays was successfully employed before [7], [14]–[17]. In addition, we introduce a second output parameter, the intensity weighted area percentage, termed ‘colony intensity percentage’. This parameter also takes the number of cells in the colonies into account. Colony intensity percentage results are useful to differentiate between wells that have a similar coverage with colonies, while containing different numbers of cells in these colonies. Thus colony area percentage may be more reflective of cell survival, while colony intensity percentage in addition also reports on the ability of the cells to grow densely, a hallmark of cancer cells.

Non-uniform illumination during scanning can lead to shading in areas of the image, which essentially corresponds to an uneven background across the image. This may lead the plugin to identify parts of the background as cells, but can be easily detected during the above mentioned visual inspection step. For such cases we have included an option that allows quantification of a sub-region of the well images. To use the sub-region analysis, after the visual inspection, the user will have to simply select a region with correct cell identification and the plugin will recalculate the results on that region. At this point the user will also be provided with the choice to reanalyze only a selection, or the entire set of wells. Results that were obtained from such a sub-region analysis can be readily compared to those obtained from the full well, as also here we calculate colony area and colony intensity percentages, however, only on the selected region. Finally, the plugin presents results in a table where every well is listed together with its colony area percentage and its colony intensity percentage.

### ColonyArea accurately identifies different inhibitory potencies of two staurosporines on glioma cell growth

**Figure 5:**
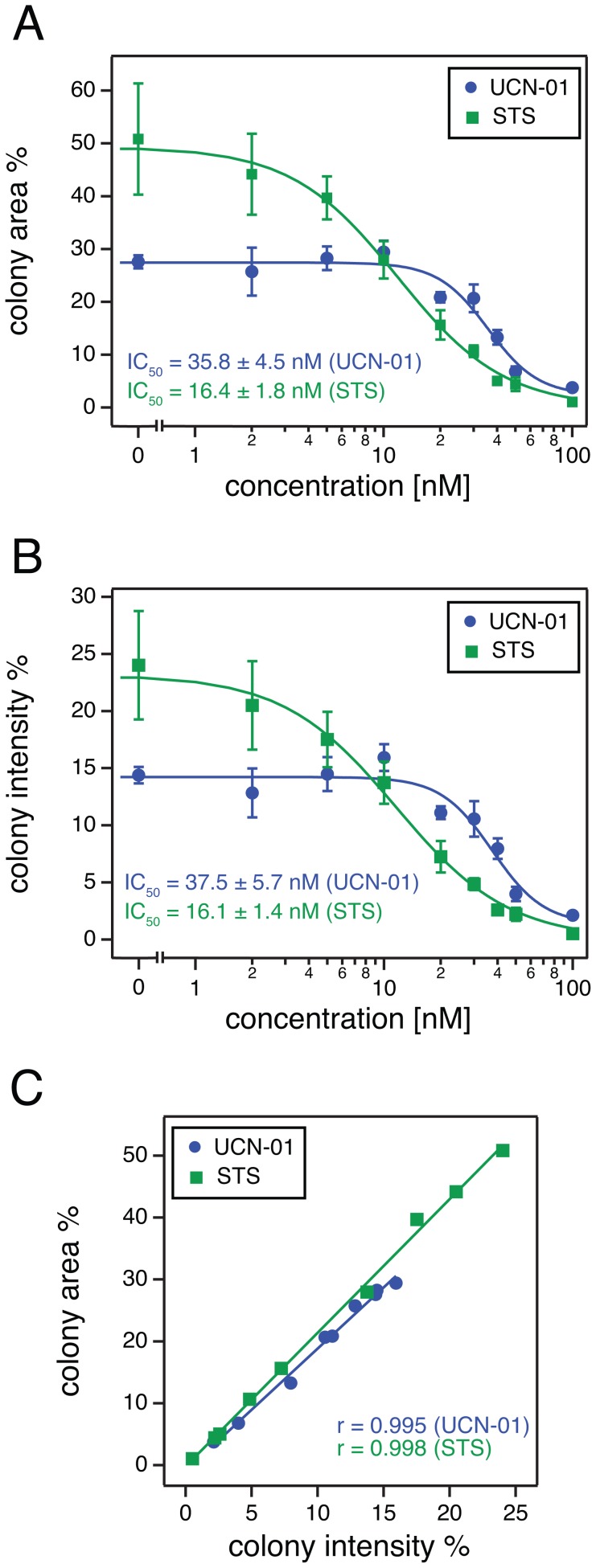
Quantification of T98G glioma cell growth after treatment with UCN-01 or staurosporine using the ColonyArea. Colony formation of T98G human glioma cells was studied after treatment with increasing concentrations of the staurosporine derivative UCN-01 or staurosporine (STS). Image data were analyzed using ColonyArea. Its output parameters were then used to generate dose response curves and determine the half maximal inhibitory concentrations (IC_50_) of the compounds. (**A**) Examples of dose response curves using the colony area percentage; IC_50_ = 35.8±4.5 nM (UCN-01) and IC_50_ = 16.4±1.8 nM (STS). (**B**) Examples of dose response curves using the colony intensity percentage; IC_50_ = 37.5±5.7 nM (UCN-01) and IC_50_ = 16.1±1.4 nM (STS). Dots correspond to averages and error bars to the standard deviations of measurements from four wells. Curves were fitted using equation (**3** **)**. Additional independent experimental repeats can be found in **Figures S1, S2, S3, S4**. (**C**) Correlation analysis of results obtained using the colony area percentage and results obtained using the colony intensity percentage. Regression lines are drawn and the Pearson product moment correlation coefficients ‘r’ is displayed for each data set.

In order to test the quantification capabilities of ColonyArea, we performed colony formation assays with the human glioma cell line T98G. We treated this cell line with increasing concentrations of two different staurosporines (UCN-01 and staurosporine), two drugs that are known to inhibit cell proliferation of this and other cell lines with slightly different potencies [18]–[22]. Staurosporines potently inhibit a wide range of serine/threonine and tyrosine protein kinases, in particular protein kinase C (PKC) [20], [21], [23]. Scanned images of 12-well plates containing treated T98G cells were analyzed using ColonyArea and dose response curves obtained from three independent biological preparations with each datapoint in quadruplicate were generated. Dose response analysis of both the colony area percentage (**Figure 5A**) and the colony intensity percentage (**Figure 5B**) allowed determining IC_50_ values for UCN-01 and staurosporine (STS). Average results from the three biological repeats (**Figure 5** and **Figures S1, S2, S3, S4**) with the corresponding standard errors of the mean (SEM) were 35.5±0.6 nM, using colony area percentage, and 37.5±0.3 nM, with colony intensity percentage, for UCN-01. The potency for STS was slightly higher, being 20.6±4.6 nM, determined using colony area percentage and 20.5±4.7 nM, from the colony intensity percentage. IC_50_ values obtained with our two quantification methods, colony area percentage and colony intensity percentage, are almost identical. This is in agreement with the excellent correlation of the respective datasets (**Figure 5C** and **Figures S1, S2, S3, S4**).

As compared to published data, our method could successfully recover the relative difference in potencies between UCN-01 and STS that were determined by others in A549 lung and MCF-7 breast carcinoma cell lines [18]. Reported IC_50_ values of UCN-01 (∼20 nM, counting cells [24]) or STS (∼3 nM, MTT-proliferation assay [20]) for their activity against T98G cells were in the same concentration regime as the ones we found. However, it can be expected that the different methods lead to different absolute results. Moreover, treatment of cells for longer times (3 days and 5 days respectively in the above publications, as compared to our 48 h) could lead to a more potent growth arrest effect [19] and therefore lower IC_50_ values.

### Direct comparison of ColonyArea measurements with an alternative colony growth quantification method

In order to validate our method, we performed a direct quantitative comparison with an alternative method by Kueng *et al.*, in which the absorption of the crystal violet dye that is washed out from stained cells is measured [9]. Thus the exact same samples that were analyzed with ColonyArea, were quantified by the absorption based method, which allowed to validate each of our measurements.

**Figure 6:**
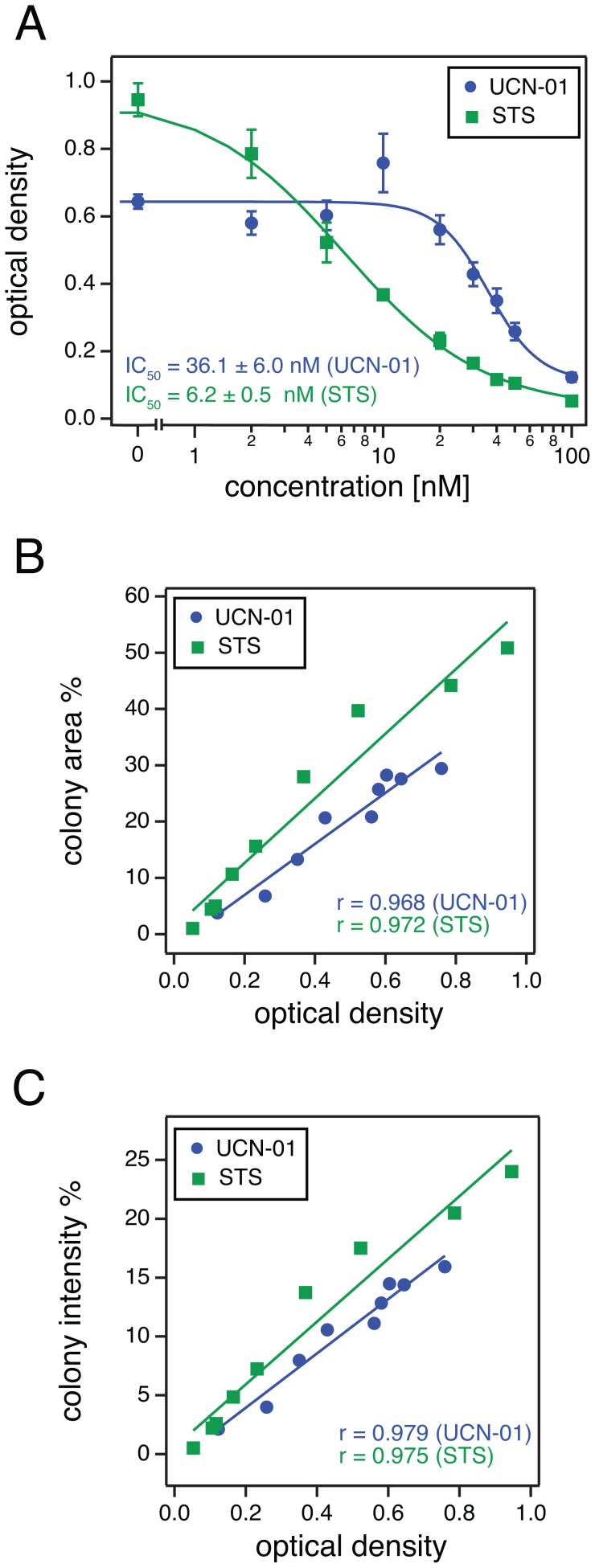
Comparison of the ColonyArea quantification with an absorption method. The identical wells that were quantified with ColonyArea in **Figure 5** were analyzed using a method where the absorption of the crystal violet dye that was washed out from labeled cells is measured. (**A**) Examples of dose response curves using the optical density from the absorbance measurements of the dye; IC_50_ = 36.1±6.0 nM (UCN-01) and IC_50_ = 6.2±0.5 nM (STS). Dots correspond to averages and error bars to the standard deviations of the exact same four wells that were analyzed in **Figure 5**. Curves were fitted using equation (**3**). (**B**) Correlation analysis of data from **Figure 5A** that were obtained using the colony area percentage and those obtained using the absorbance in (**A**). (**C**) Correlation analysis of colony intensity percentage from **Figure 5B** and the corresponding absorbance data. Regression lines are drawn and the Pearson product moment correlation coefficients ‘r’ is displayed for each set of data. Additional correlative analysis of experimental repeats can be found in **Figures S1, S2, S3, S4**.

Using the absorption method we obtained average IC_50_ values of 30.5±2.8 nM for UCN-01 and 9.3±1.8 nM for STS (three independent biological repeats, SEM). Therefore, the absolute differences between the IC_50_ values of UCN-01 and STS, which were obtained with either our plugin (**Figure 5A,B**) or with Kueng’s method (**Figure 6A**), are in very good agreement (**Table 1**). The lower values found for the absorbance derived data may for example be due to incomplete dissolution of the dye from the cells.

**Table 1:** IC_50_ values of staurosporines as determined from imaging (ColonyArea) and absorption based methods.

	area %	intensity %	optical density
UCN-01	35.5±0.6	37.5±0.3	30.5±2.8
STS	20.6±4.6	20.5±4.7	9.3±1.8

IC_50_ values derived from the ColonyArea output parameters colony area percentage (area %), colony intensity percentage (intensity %) and from the absorption based method (optical density) are given in nM for the two compounds UCN-1 and staurosporine (STS).

Average Pearson product moment correlation coefficient from all our biological repeats (**Figure 6B,C** and **Figures S1, S2, S3, S4**) show a high level of correlation between our plugin results and Kueng's absorption based method (r = 0.94 for colony area percentage and r = 0.95 for colony intensity percentage), which validates our approach.

Therefore, our imaging based method is as accurate as an alternative colony growth quantification procedure, while it preserves the samples for documentation and reevaluation. It furthermore performs an automatic quantification without the need of expensive equipment like a plate reader or a spectrophotometer.

An indirect comparison between our method and the ImageJ macros provided by Cai and coworkers [4] shows that our method requires about 1/5 of the time that their method requires. Cai and coworkers mention that for a typical experiment with 30 wells of colonies they require about 60 minutes [4], while in our case for a similar sample less than 10 minutes are required for scanning and image analysis.

## Conclusions

We have presented the ImageJ plugin ColonyArea, which facilitates quantification of colony formation assays from scanned well plate images. The plugin circumvents typical image analysis problems associated with the actual counting of cell colonies, as it quantifies the percentage of the well area that is covered with cells. We also derived an intensity weighted area percentage that in addition incorporates the cell density proportional staining intensity. Automation and standardization eliminate experimenter dependent variations associated with manual counting of colonies. Like all other ImageJ plugins, this plugin is available freely for download. We hope that the plugin in combination with the simple and inexpensive raw data acquisition will be of broad use in many laboratories for routine and parallelized analysis applications.

## Methods

### Cell culture

Human glioblastoma T98G cells (VTT Technical Research Centre, Turku, Finland) [25] were cultured in Eagle's Minimum Essential Media (EMEM) (Sigma-Aldrich, St. Louis MI - USA) supplemented with 10% heat-inactivated FCS, 2 mM L-glutamine and penicillin (100 units/mL)–streptomycin (100 units/mL) in a humidified atmosphere of 5% CO_2_ at 37°C.

### Colony formation assay

2500 cells per well were plated in 12-well plates (Greiner Bio One Cellstar, Frickenhauser - Germany) and were allowed to grow for about 4 to 5 days until small colonies could be clearly seen. Cells were treated for 48 hrs with different concentrations (2–100 nM) of staurosporine or UCN-01 (7-hydroxystaurosporine) in growth media. For each concentration datapoint of the two drugs, cells were analyzed in quadruplicates. Staurosporine was purchased as 1 mM ready-made solution in DMSO (Sigma Cat # S6942) and UCN-01 as powder (Sigma Cat # U6508). UCN-01 was diluted in DMSO according to the manufacturer's instructions. Cell culture plates containing colonies were gently washed with PBS and fixed with 3.7% formaldehyde for 10 minutes. Wells were rinsed once again with PBS and colonies were stained with 0.2% crystal violet solution in 10% ethanol for 10 minutes. Excess stain was removed by washing repeatedly with PBS. All the procedures were done at room temperature. The plates can be stored at room temperature or at +4 °C for several months without any visible fading of the dye.

### Quantification of colony formation using the ColonyArea plugin

The colony area percentage was calculated on thresholded and intensity inverted regions that were by default single well images or alternatively user-selected regions within a well (**Figure 4B**), as:
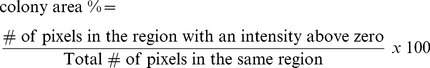
(1)


To calculate the colony intensity percentage our plugin uses the following formula:
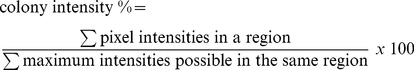
(2)


Here 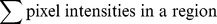, represents the added intensity of all pixels identified as belonging to cells inside the region and 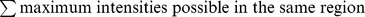, represents the sum of all the well-pixels within the same region of interest multiplied by 255, i.e. assuming highest intensity with full saturation of these pixels.

### Quantification of colony formation measuring absorption

Colony formation was quantified following the method described by Kueng and coworkers [9]. In brief, the crystal violet staining of cells from each well was solubilized using 1 ml of 10% acetic acid and the absorbance (optical density) of the solution was measured on a Synergy H1 hybrid fluorescence plate-reader (BioTek, Winooski, VT, USA) at a wavelength of 590 nm.

### Dose response curves

After successful quantification of the colony formation, we determined the half maximal inhibitory concentrations, IC_50_, of the compounds from dose response curves. Dose response curves from three different parameters: colony area percentage and colony intensity percentage, both obtained with our plugin, and the absorption of the crystal violet dye [9] were generated and compared. Plots and IC_50_ values were obtained using the freely available dose response package “DRC package” developed by Christian Ritz and Jens Strebing [26] which runs on the free software programming language R (R Development Core Team, Vienna, Austria).

Dose response data were fitted using the four-parameter log-logistic function of the DRC package:
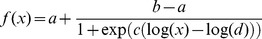
(3)where 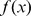 is the parameter quantifying the colony formation, i.e. either the colony area percentage, the colony intensity percentage or the absorption and 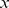 is the compound concentration. The fitting parameters 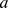 and 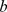 correspond to the lower and upper limits respectively, 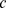 to the steepness of the increase and 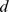 corresponds to the IC_50_ value.

### Correlation analysis

Correlation analysis of colony area precentage, colony intensity percentage and absorbance derived data was done using IGOR Pro 6 (WaveMetrics, Tigard, OR, USA). Pearson product moment correlation coefficients were calculated and displayed together with the regression lines of each pair of data.

### Colony formation assay image acquisition

The plugin requires high quality images of more than 800 dpi, preferably 1200 dpi or higher. Here, images were acquired using a flatbed scanner Epson perfection V700 (Epson, Nagano – Japan) using the following settings: 24-bit colour, 1200 dpi resolution and an unsharp mask filter to make edges as clear and distinct as possible. No backlight correction was applied. Plates were placed in the center of the scanner and covered with the white background lid of the scanner to allow for a uniform illumination. Images should be acquired such that the background is light colored and the cells are dark. Files were saved in the tagged image file format (Tiff). For further details, see the user manual of ColonyArea provided in the installation package of the plugin. This plugin has been conceived to work only on standard rectangular cell culture plates with circular wells distributed evenly.

### ImageJ requirements for the ColonyArea plugin and download bundle

This plugin requires an ImageJ version that includes the java compiler, version1.47n or later. For convenience ColonyArea plugin comes as a packaged bundle, containing the files Colony_area.java and Colony_area.class that represent the actual plugin in the stricter sense, as well as the macros Colony_thresholder.ijm, Manual_colony_thresholder.ijm and Colony_measurer.ijm that execute some functions of the plugin. In addition, a user manual file, ColonyArea_manual.pdf that provides all the necessary details for operation and a file, gpl.rtf that contains the GNU general public license of this plugin are included. The ColonyArea plugin bundle can be found as **Information S1**, accompanying this paper or on the webpage from our group http://www.btk.fi/research/research-groups/abankwa/downloads/ and the webpage of the European Data Infrastructure (EUDAT) https://b2share.eudat.eu/record/45 through their service B2SHARE. On this webpages the reader can also find a set of sample images that were used to obtain the results in **Figure S2**.

## Supporting Information

Figure S1 **Second independent repeat of ColonyArea and absorbance based analysis of T98G glioma cell survival and growth after treatment with UCN-01 and comparison of the data obtained with the two methods.** Colony formation analysis of T98G human glioma cells after treatment with increasing concentrations of the staurosporine derivative UCN-01. Dose response curves derived from (**A**) the colony area percentage giving an IC_50_ = 34.4±2.9 nM; (**B**) the colony intensity percentage giving an IC_50_ = 37.1±7.0 nM; or (**C**) the optical density of the washed out crystal violet dye giving an IC_50_ = 27.2±3.3 nM. Dots correspond to averages and error bars to the standard deviations of four replica samples. Curves were fitted using equation (**3**). (**D**–**F**) Correlation analysis between pairs of data presented in **A–C**, as indicated on the axes. Regression lines are drawn and the Pearson product moment correlation coefficients ‘r’ is displayed for each data comparison.
(PDF)

Figure S2 **Third independent repeat of ColonyArea and absorbance based analysis of T98G glioma cell survival and growth after treatment with UCN-01 and comparison of the data obtained with the two methods.** Colony formation analysis of T98G human glioma cells after treatment with increasing concentrations of the staurosporine derivative UCN-01. Dose response curves derived from (**A**) the colony area percentage giving an IC_50_ = 36.4±4.0 nM; (**B**) the colony intensity percentage giving an IC_50_ = 38.0±9.7 nM; or (**C**) the optical density of the washed out crystal violet dye giving an IC_50_ = 28.2±4.4 nM. Dots correspond to averages and error bars to the standard deviations of four replica samples. Curves were fitted using equation (**3**). (**D**–**F**) Correlation analysis between pairs of data presented in **A–C**, as indicated on the axes. Regression lines are drawn and the Pearson product moment correlation coefficients ‘r’ is displayed for each data comparison.
(PDF)

Figure S3 **Second independent repeat of ColonyArea and absorbance based analysis of T98G glioma cell survival and growth after treatment with staurosporine (STS) and comparison of the data obtained with the two methods.** Colony formation analysis of T98G human glioma cells after treatment with increasing concentrations of staurosporine. Dose response curves derived from (**A**) the colony area percentage giving an IC_50_ = 27.3±3.7 nM; (**B**) the colony intensity percentage giving an IC_50_ = 27.4±6.2 nM; or (**C**) the optical density of the washed out crystal violet dye giving an IC_50_ = 9.5±2.0 nM. Dots correspond to averages and error bars to the standard deviations of four replica samples. Curves were fitted using equation (**3**). (**D**–**F**) Correlation analysis between pairs of data presented in **A–C**, as indicated on the axes. Regression lines are drawn and the Pearson product moment correlation coefficients ‘r’ is displayed for each data comparison.
(PDF)

Figure S4 **Third independent repeat of ColonyArea and absorbance based analysis of T98G glioma cell survival and growth after treatment with staurosporine (STS) and comparison of the data obtained with the two methods.** Colony formation analysis of T98G human glioma cells after treatment with increasing concentrations of staurosporine. Dose response curves derived from (**A**) the colony area percentage giving an IC_50_ = 22.6±0.5 nM; (**B**) the colony intensity percentage giving an IC_50_ = 22.5±2.1 nM; or (**C**) the optical density of the washed out crystal violet dye giving an IC_50_ = 12.3±5.7 nM. Dots correspond to averages and error bars to the standard deviations of four replica samples. Curves were fitted using equation (3). (**D–F**) Correlation analysis between pairs of data presented in **A–C**, as indicated on the axes. Regression lines are drawn and the Pearson product moment correlation coefficients ‘r’ is displayed for each data comparison.
(PDF)

Information S1 **The additional file ‘ColonyArea.zip’ contains the plugin installation files and user manual.** In this zip-compressed file, we provide seven plugin files: Colony_area.java; Colony_area.class; Colony_thresholder.ijm; Manual_colony_thresholder.ijm; Colony_measurer.ijm; a user manual file, ColonyArea_manual.pdf and; the GNU general public license, gpl.rtf.
(ZIP)
