# Supplementary figures and images for: ColonyArea: An ImageJ Plugin to Automatically Quantify Colony Formation in Clonogenic Assays
Source: PLoS One. 2014 Mar 19;9(3):e92444. doi: 10.1371/journal.pone.0092444 (PMC3960247; doi:10.1371/journal.pone.0092444)

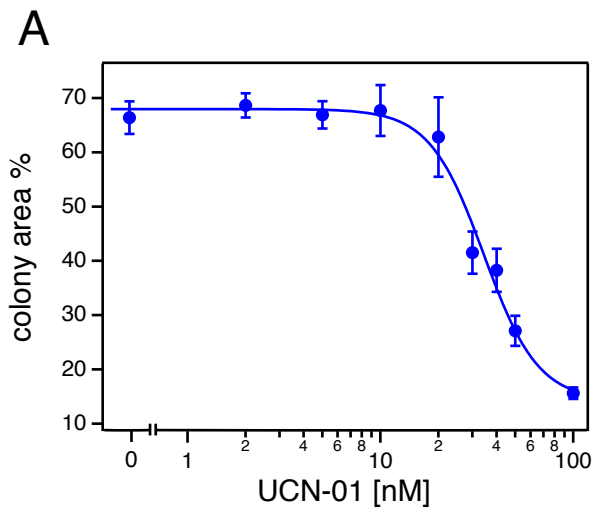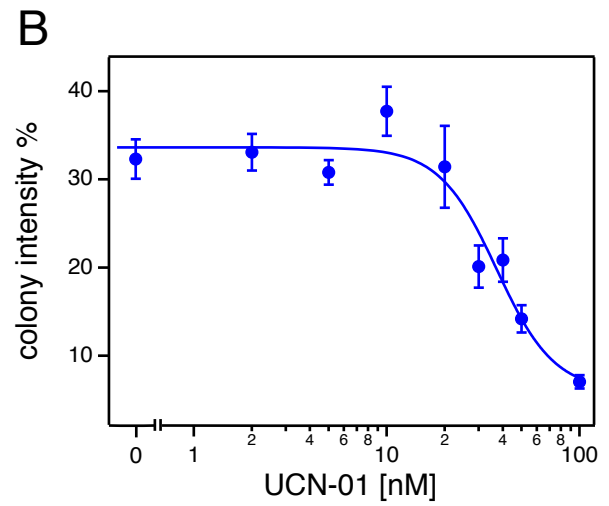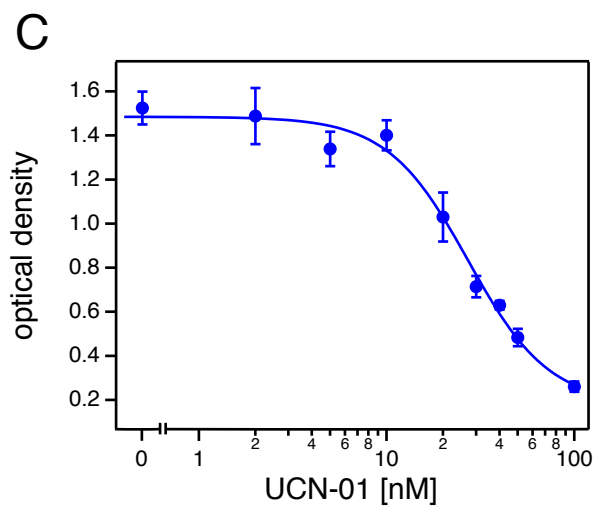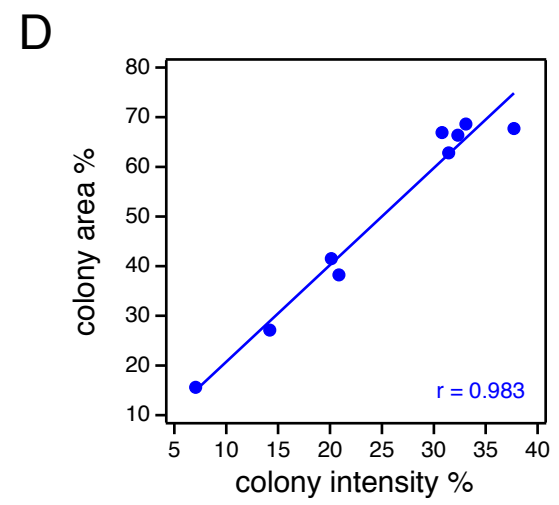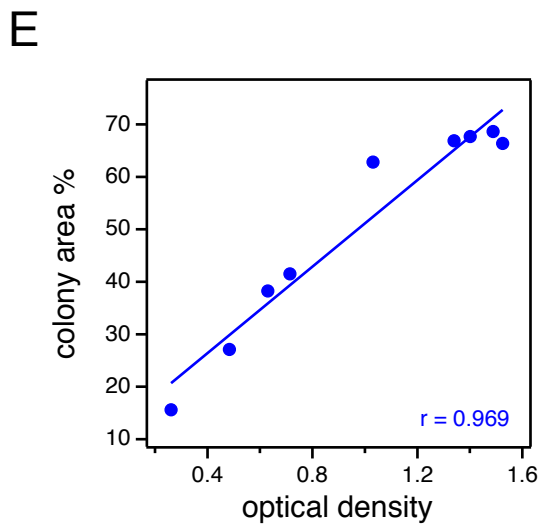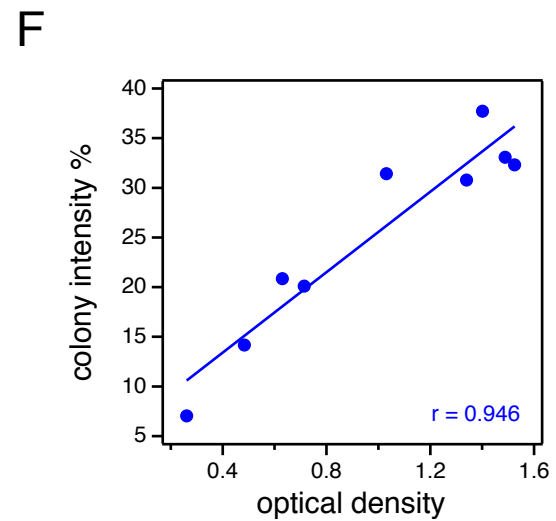

Supplement: Figure S1 — Second independent repeat of ColonyArea and absorbance based analysis of T98G glioma cell survival and growth after treatment with UCN-01 and comparison of the data obtained with the two methods. Colony formation analysis of T98G human glioma cells after treatment with increasing concentrations of the staurosporine derivative UCN-01. Dose response curves derived from (A) the colony area percentage giving an IC50 = 34.4±2.9 nM; (B) the colony intensity percentage giving an IC50 = 37.1±7.0 nM; or (C) the optical density of the washed out crystal violet dye giving an IC50 = 27.2±3.3 nM. Dots correspond to averages and error bars to the standard deviations of four replica samples. Curves were fitted using equation (3 ). (D–F) Correlation analysis between pairs of data presented in A–C, as indicated on the axes. Regression lines are drawn and the Pearson product moment correlation coefficients ‘r’ is displayed for each data comparison. (PDF) [file pone.0092444.s001.pdf]

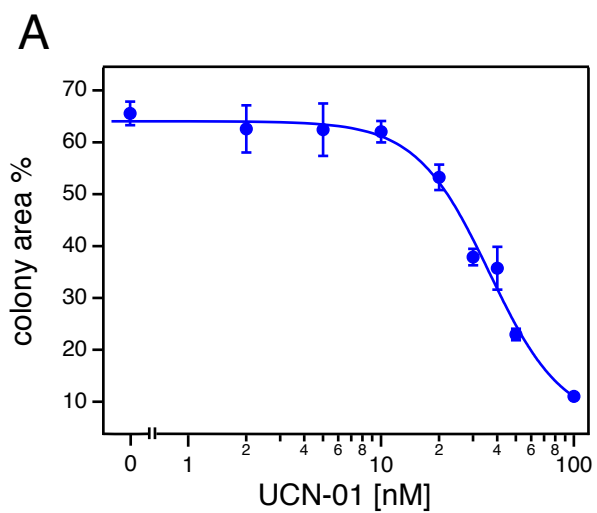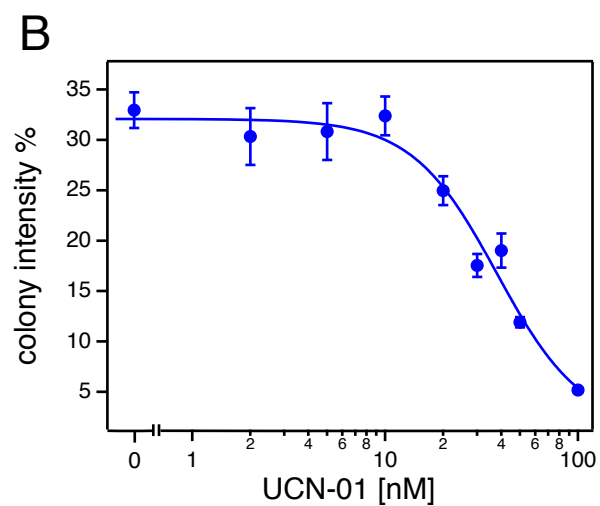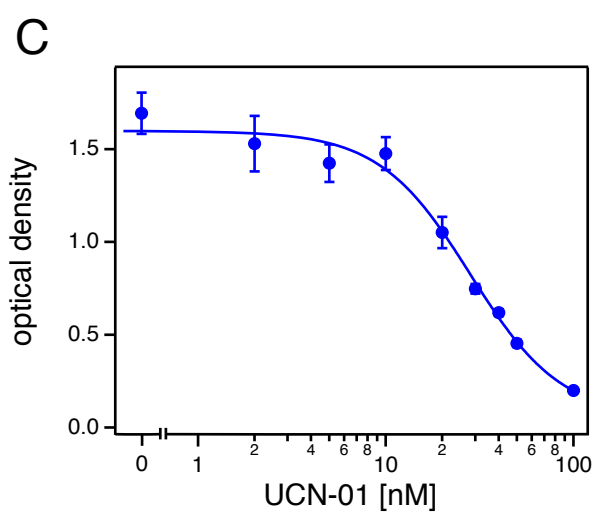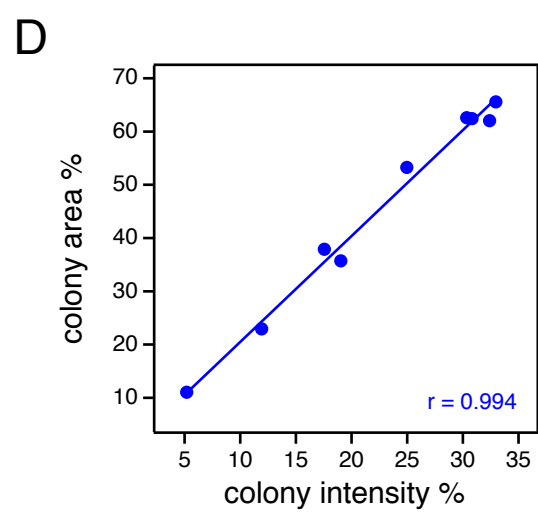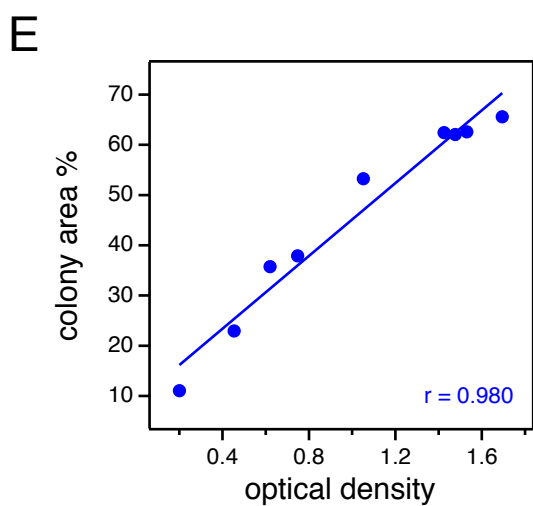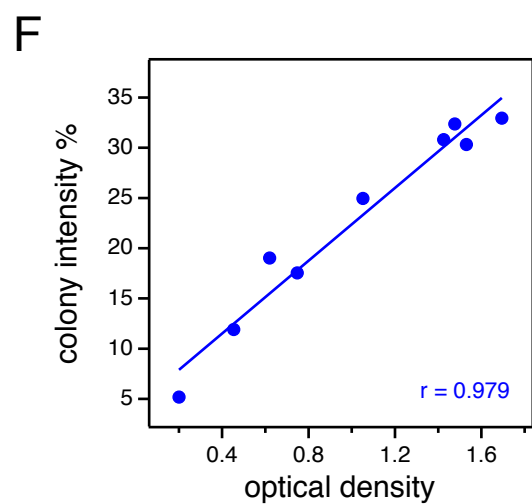

Supplement: Figure S2 — Third independent repeat of ColonyArea and absorbance based analysis of T98G glioma cell survival and growth after treatment with UCN-01 and comparison of the data obtained with the two methods. Colony formation analysis of T98G human glioma cells after treatment with increasing concentrations of the staurosporine derivative UCN-01. Dose response curves derived from (A) the colony area percentage giving an IC50 = 36.4±4.0 nM; (B) the colony intensity percentage giving an IC50 = 38.0±9.7 nM; or (C) the optical density of the washed out crystal violet dye giving an IC50 = 28.2±4.4 nM. Dots correspond to averages and error bars to the standard deviations of four replica samples. Curves were fitted using equation (3 ). (D–F) Correlation analysis between pairs of data presented in A–C, as indicated on the axes. Regression lines are drawn and the Pearson product moment correlation coefficients ‘r’ is displayed for each data comparison. (PDF) [file pone.0092444.s002.pdf]

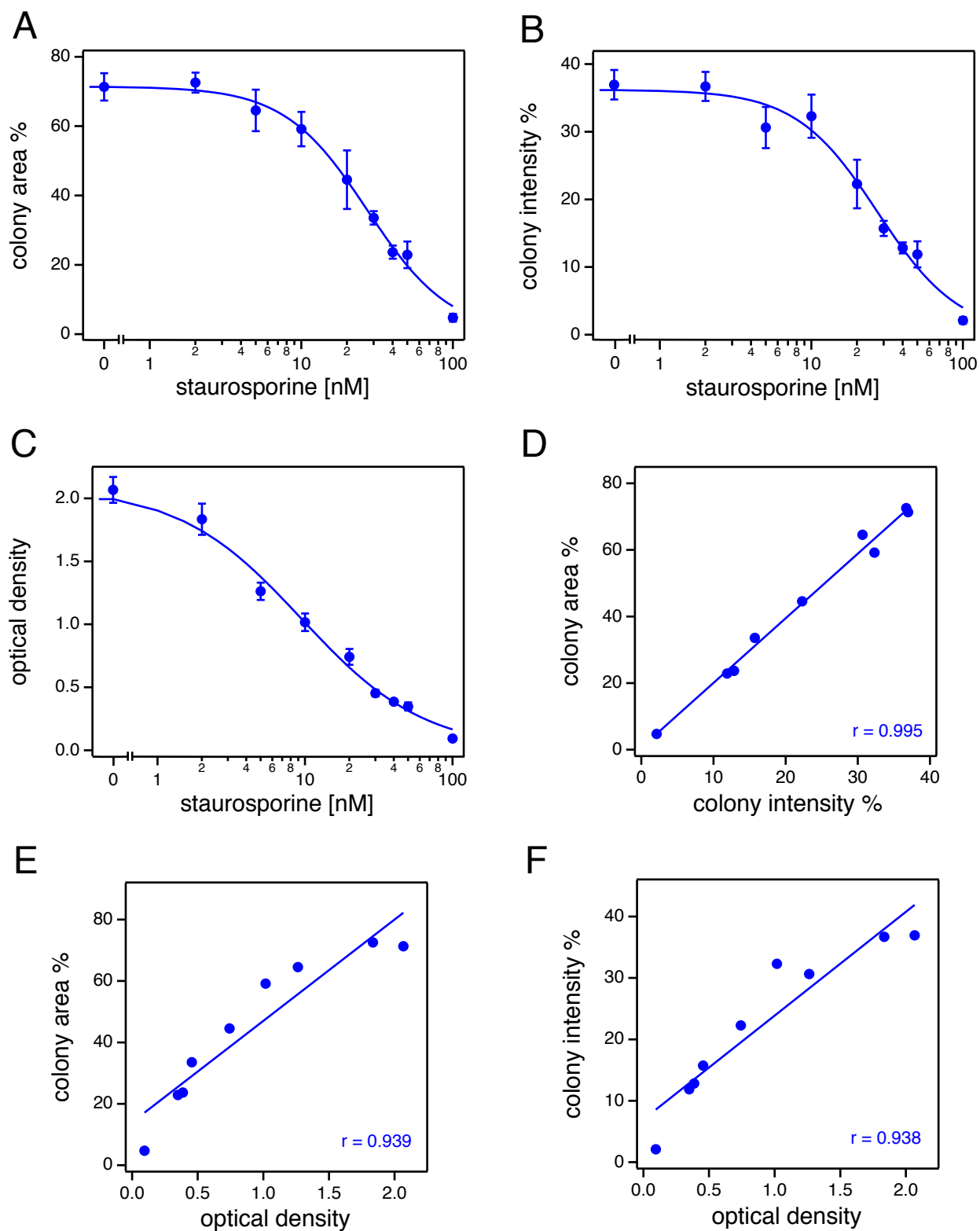

Supplement: Figure S3 — Second independent repeat of ColonyArea and absorbance based analysis of T98G glioma cell survival and growth after treatment with staurosporine (STS) and comparison of the data obtained with the two methods. Colony formation analysis of T98G human glioma cells after treatment with increasing concentrations of staurosporine. Dose response curves derived from (A) the colony area percentage giving an IC50 = 27.3±3.7 nM; (B) the colony intensity percentage giving an IC50 = 27.4±6.2 nM; or (C) the optical density of the washed out crystal violet dye giving an IC50 = 9.5±2.0 nM. Dots correspond to averages and error bars to the standard deviations of four replica samples. Curves were fitted using equation (3 ). (D–F) Correlation analysis between pairs of data presented in A–C, as indicated on the axes. Regression lines are drawn and the Pearson product moment correlation coefficients ‘r’ is displayed for each data comparison. (PDF) [file pone.0092444.s003.pdf]

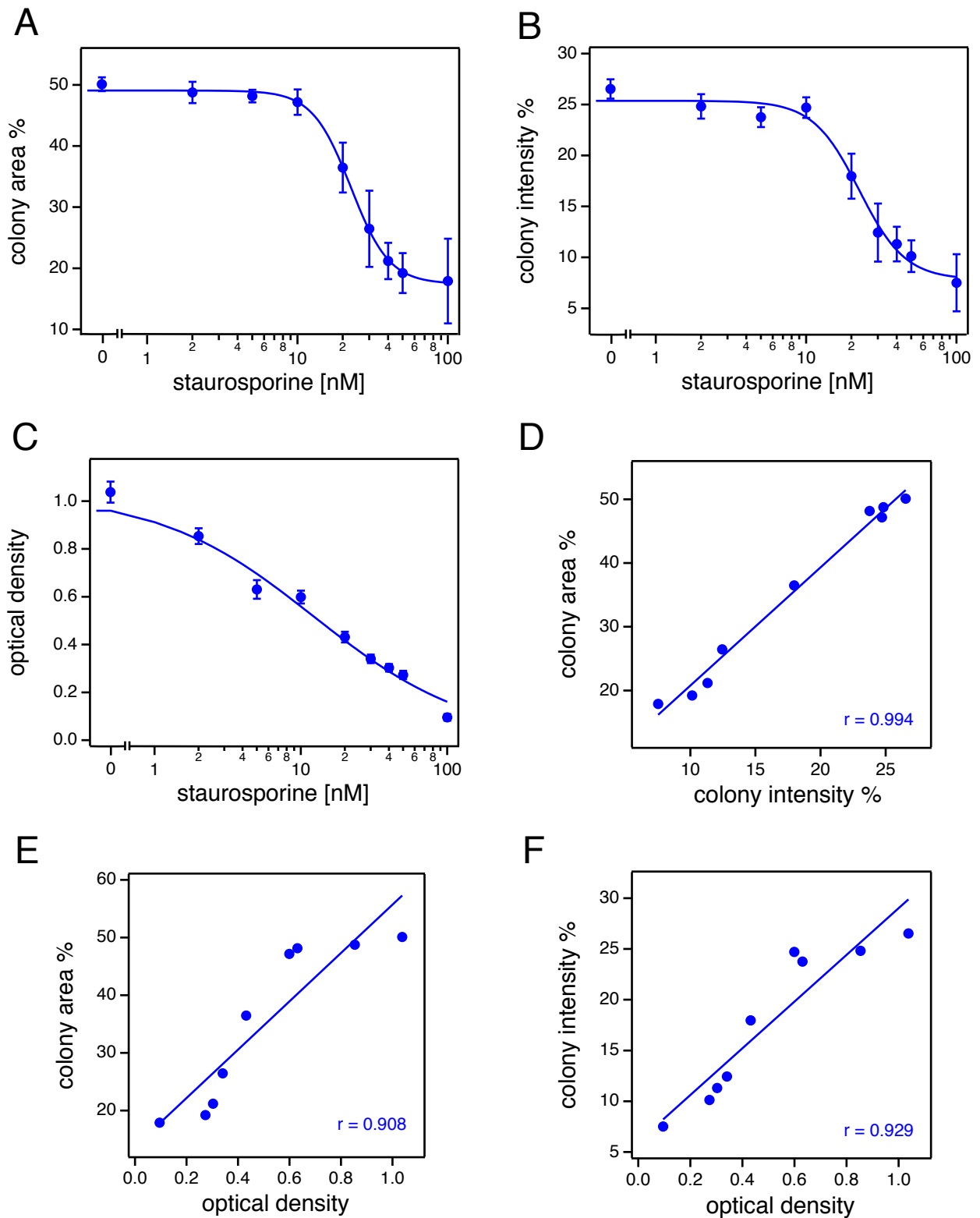

Supplement: Figure S4 — Third independent repeat of ColonyArea and absorbance based analysis of T98G glioma cell survival and growth after treatment with staurosporine (STS) and comparison of the data obtained with the two methods. Colony formation analysis of T98G human glioma cells after treatment with increasing concentrations of staurosporine. Dose response curves derived from (A) the colony area percentage giving an IC50 = 22.6±0.5 nM; (B) the colony intensity percentage giving an IC50 = 22.5±2.1 nM; or (C) the optical density of the washed out crystal violet dye giving an IC50 = 12.3±5.7 nM. Dots correspond to averages and error bars to the standard deviations of four replica samples. Curves were fitted using equation (3). (D–F) Correlation analysis between pairs of data presented in A–C, as indicated on the axes. Regression lines are drawn and the Pearson product moment correlation coefficients ‘r’ is displayed for each data comparison. (PDF) [file pone.0092444.s004.pdf]
