# Supplementary material for: ColonyArea: An ImageJ Plugin to Automatically Quantify Colony Formation in Clonogenic Assays
Source: PLoS One. 2014 Mar 19;9(3):e92444. doi: 10.1371/journal.pone.0092444 (PMC3960247; doi:10.1371/journal.pone.0092444)
Supplement: Information S1 — The additional file ‘ColonyArea.zip’ contains the plugin installation files and user manual. In this zip-compressed file, we provide seven plugin files: Colony_area.java; Colony_area.class; Colony_thresholder.ijm; Manual_colony_thresholder.ijm; Colony_measurer.ijm; a user manual file, ColonyArea_manual.pdf and; the GNU general public license, gpl.rtf. (ZIP) [file pone.0092444.s005.zip › ColonyArea/ColonyArea User Manual.pdf]

User Manual for the ImageJ plugin: “ColonyArea”

Manish Bagga, Camilo Guzmán and Daniel Abankwa

Introduction

Purpose of the plugin:

We present an ImageJ plugin that precisely and rapidly quantifies colony formation assays in different sizes of cell culture plates. The plugin automatically separates all the wells present in the imaged plate, and quantifies the percentages of area that is covered by colonies. This plugin also provides information about the intensity percentage taking into consideration not only the area covered by the colonies, but also the intensity of staining as a direct relation to the number of cells in a colony. By separating all the wells and making a stack of images, our plugin allows a more precise selection of the colonies (thresholding), and automates and standardizes a process that done manually will take large amounts of the valuable time of researchers.

How to install:

1. The bundle is freely available for download from our website http://www.btk.fi/research/research-groups/abankwa/downloads/.
2. Make sure that you have the latest version of ImageJ. It should be ImageJ version1.47n or later that includes the java compiler. It is highly recommended to download the latest version of ImageJ from here: http://rsbweb.nih.gov/ij/download.html. The plugin works both on the 64 or 32 bit versions of ImageJ.
3. If ImageJ is open, close it.
4. Copy the "ColonyArea" folder with its 6 files into the "Plugins" folder of the ImageJ installation folder (See "Files contained in the plugin" for details).
5. Open ImageJ.
6. Depending on factors such as the image resolution and size, ImageJ might need more RAM memory than what is given on its default settings. 650 MB should be sufficient for most calculations but if you notice delays or the plugin is non-responsive you can change those settings.
7. To increase the amount of RAM available to ImageJ:
   - Go to Edit→Options→Memory& Threads.
   - Give it NO more than 75% of the total RAM on the working computer. Using more than 75% could result in virtual RAM being used, which may cause ImageJ to become slow and unstable.

Image Requirements:

1. We recommend that images to be processed should have a resolution higher than 800 dpi, preferably higher than 1200 dpi. The plugin would work on images with lower resolution too, but in such cases the image quality would be very low and the reliability of the measurement might become compromised.
2. Images should be preferably in a ".tiff" format because this format allows for a higher resolution and image quality. Formats like ".png", ".jpeg", ".bmp",

".gif" are also supported but our tests show that results are not as accurate as with ".tiff" due to the lower image quality in those formats.

I. Image straightening and ROI selection:

1. Close all images opened in ImageJ and open the ".tiff" image of the colony formation assay that you want to process.
2. Go to Image→Transform→Rotate
3. Set the angle to 0, gridlines to anywhere from 20-100 depending on image size and your discretion. Tick "Preview" (See **Fig. 1**).

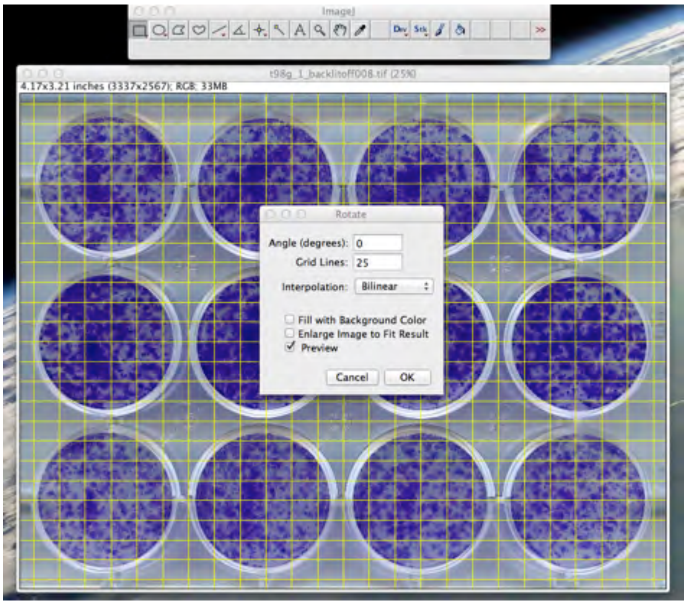

Figure 1: Use the rotate command to straighten the image.

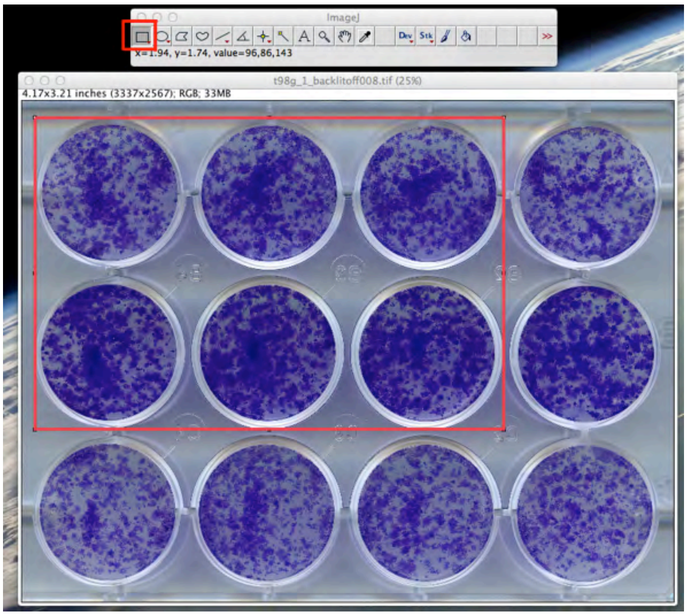

Figure 2: Use the rectangular selection tool to choose the wells to be analyzed.

4. Based on visual inspection, select an angle such that the image is nearly horizontal. (With an acceptable error of about 1-1.5 degrees max.) Then click on “OK”.
5. After the image has been straightened, use the “Rectangular Selection” tool from the ImageJ toolbar to make a selection of a region of interest (ROI) containing the wells you want to process (See **Fig. 2**). The selection should be done in such a way that the sides of the rectangle touch the outer walls of the wells. This option allows the user to analyze only a subsection of the plate if desired.

II. Thresholding and colony quantification using Colony_area:

1. After you have completed the “Image straightening and ROI selection” steps, go to the plugins menu and select “Colony_area” (Go to Plugins→ColonyArea→Colony area).
2. The plugin will ask you to choose the desired postfix for all the names of processed images and result files, plus the directory where you want every resultant file to be saved. So, a save file dialog will appear (See **Fig. 3**).

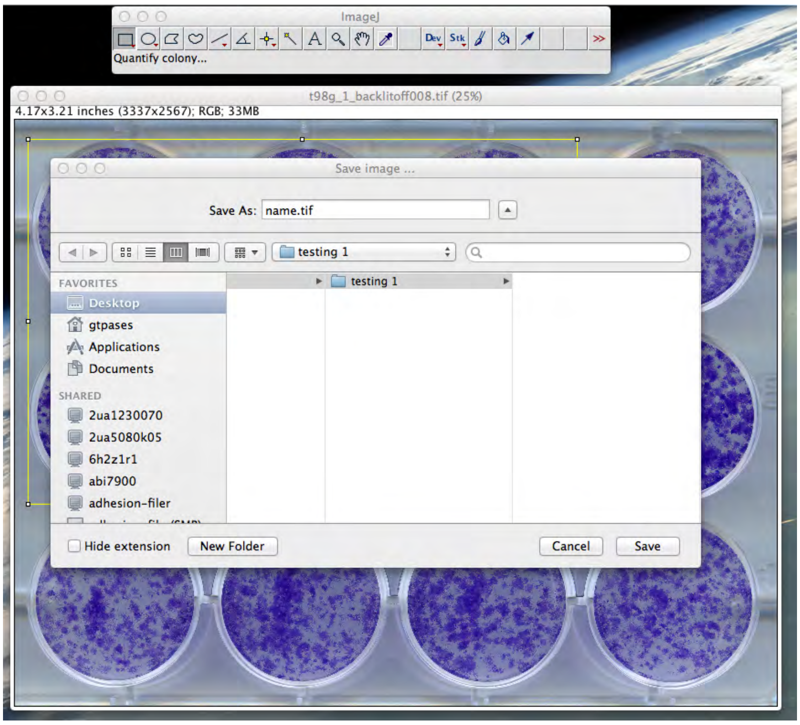

Figure 3: Choosing the location the directory for the result files and the postfix that the files will have.

3. The default for postfix is set as the name of your image file (i.e. a file called “name.tiff” will have “***_name***” as postfix).

4. The ROI will be converted to gray scale (8-bit). Then you will be presented with an option menu where you can choose the type of plate that is going to be processed (different plate sizes have different distances between wells). This choice refers only to the type of plate (6, 12 or 24-well) and not to the actual number of wells that you have selected in the ROI (See **Fig. 4**). It is also possible to use custom plates, see the “Appendix A6” of this manual for details.

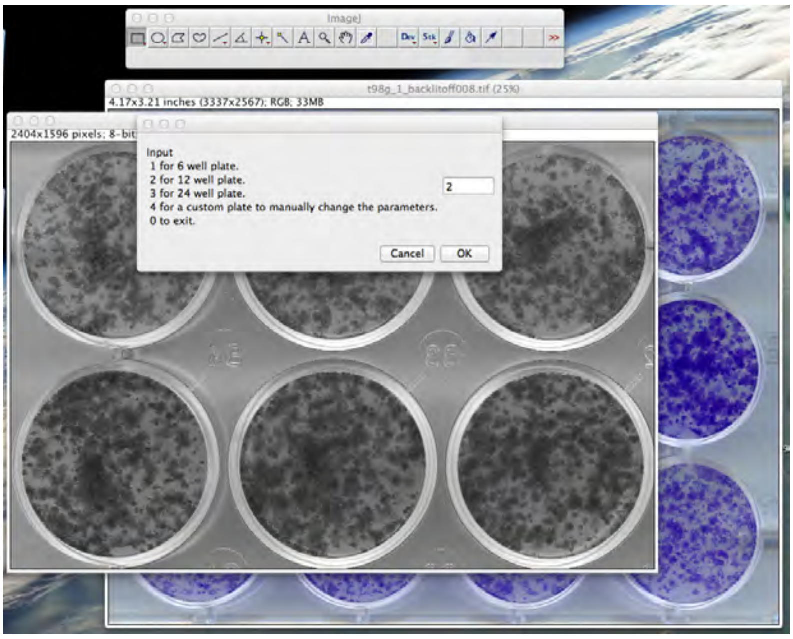

Figure 4: Operating the plugin: choose the plate type.

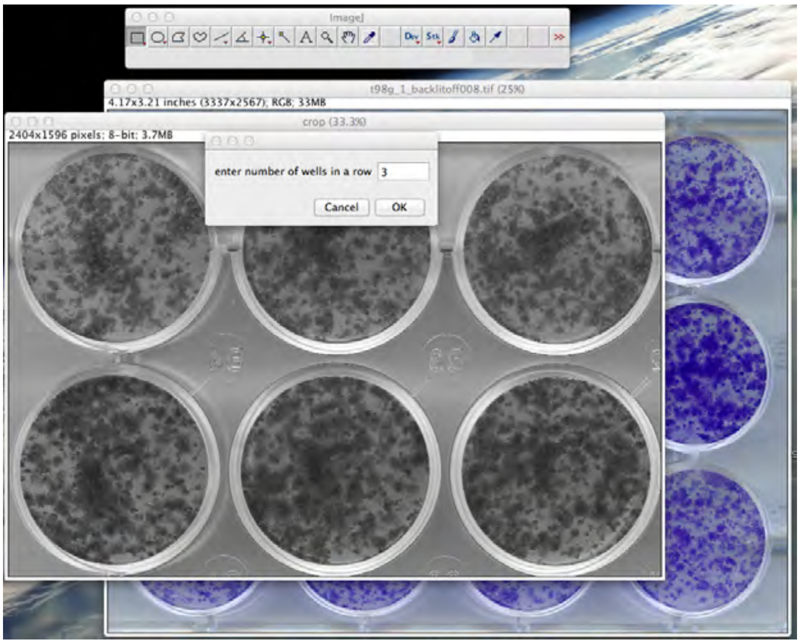

Figure 5: Operating the plugin: choose the number of wells in a row and in a column.

5. The user will be asked to give the number of wells in a row and in a column. The values provided here must correspond to the wells that you have selected in the ROI (See **Fig. 5**).

6. The plugin will proceed to crop the image, detect the wells, and create an image stack type ".tiff" of the wells. This image stack is named "wells_***name***.tiff" and it will be saved in the results folder you specified (See "Appendix" for details on file naming). The wells in this image stack are ordered starting from the well on the top-right corner of the selected region of interest (See **Fig. 6**), continuing with those wells to the right, and after proceeding with the next row in the same way (i.e. like writing a text, left to right and top to bottom). This image stack file contains only the identified wells in an 8-bit grey scale format.
   [**Note**: If the plugin detects that you have not cropped the image to a required degree of accuracy, it will ask you to correct this. See the "Appendix" of this manual for details about such case.]

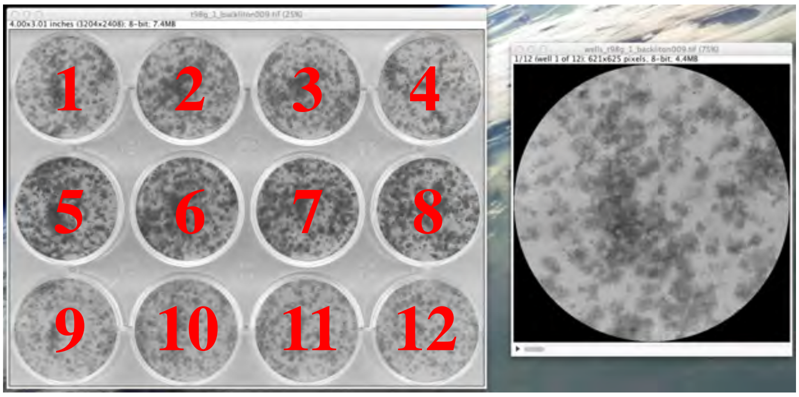

Figure 6: Example from a 12-well plate showing how the wells are ordered in the stack file ("wells_***name***.tiff"). The numbers in red have been superposed here for explanation purposes but are not present on the execution of the plugin.

7. In the next step, the image stack of the cropped and selected wells ("wells_***name***.tiff") is thresholded to detect the pixels containing the cells and remove the background. A second image stack named "thresholded_wells_***name***.tiff" will appear showing the identified colonies with their respective intensity using a "fire" lookup table (LUT) on a white background (See **Fig. 7**). Another window is also displayed with the threshold value applied to each well. This file will be saved with the name "applied_threshold_wells_***name***.txt" (See **Fig. 8**).
   [Note: There might be cases when the thresholds can not be detected according to the standard method or the result is not satisfactory, i.e. colonies are missing or background is also selected, for further explanation on how to deal with such cases see the "Appendix" section of this manual.]

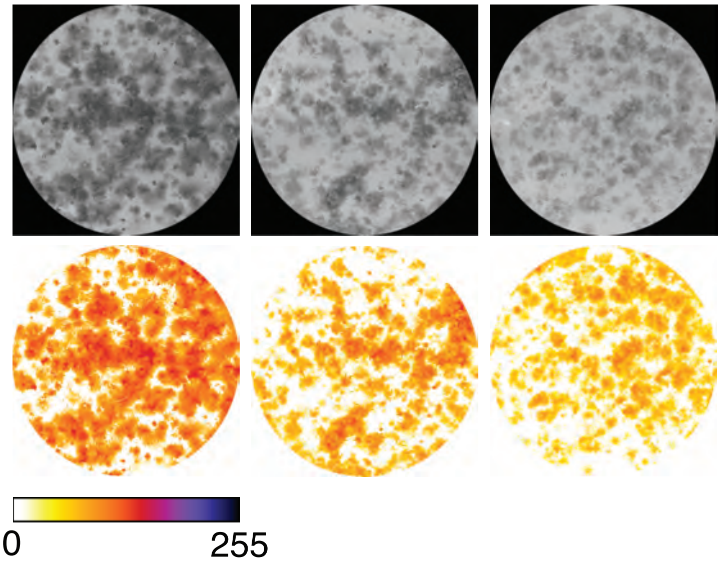

Figure 7: Examples of images contained in the two stacks that the plugin creates. Upper row shows the cropped wells (“wells_***name***.tiff”) displayed on an 8-bit grey scale. Lower row shows the identified colonies (“thresholded_wells_***name***.tiff”) with their respective intensity using a “fire” lookup table.

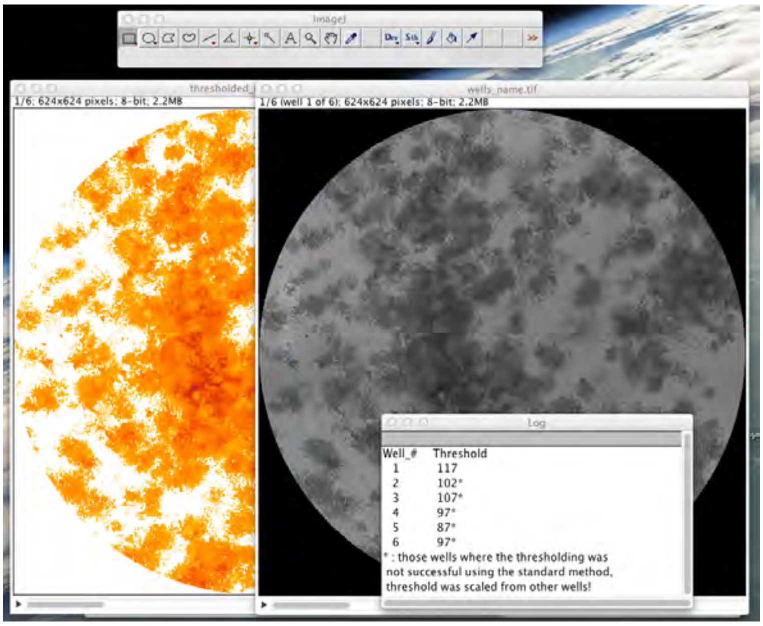

Figure 8: The plugin displays the cropped wells stack and the thresholded stack alongside to allow for visual comparison and identification of cases when parts of the background might have been selected as colonies. It also displays the computed thresholds.

8. To calculate the results use the “Colony measurer” tool. Select the window containing the thresholded wells stack (“thresholded_wells_***name***.tiff”) and go to Plugins→ColonyArea→Colony measurer (See **Fig. 9**).

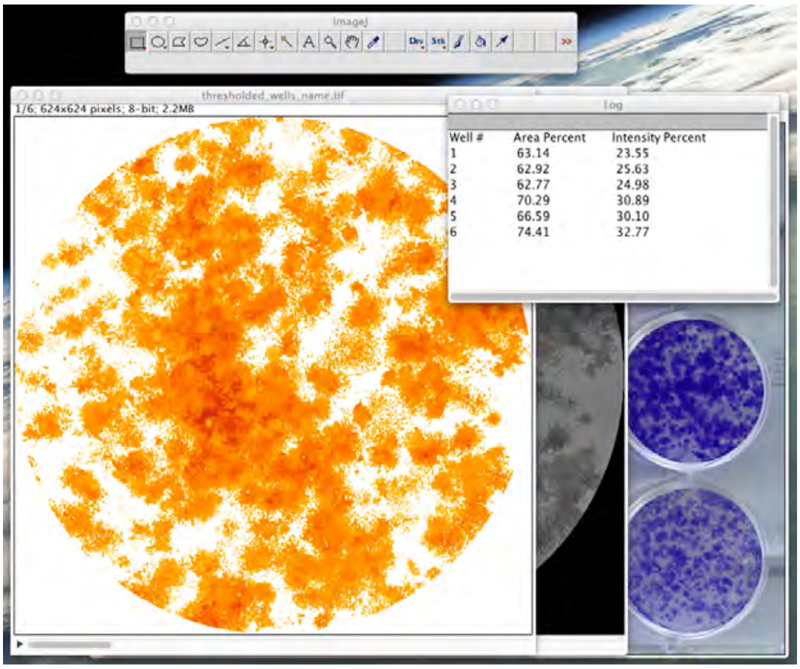

Figure 9: Run the "Colony measurer" tool to measure the area percent and intensity percent.

9. A table of results will appear in a new window and it will be saved with the name "results_thresholded_wells_***name***.txt". The columns in the table correspond to the well number, percentage of area in the well covered by the colonies "colony area percentage", and another parameter called "colony intensity percentage". Colony intensity percentage is a parameter that takes into account both the area covered by the colonies and the density of the colonies (intensity of staining for each pixel). It can be used as an independent parameter or it can be used to distinguish two or more plates that have the same cell area but have different amount of cells due to a difference in the density of colonies present. On this table each column is separated by a fixed width (number of spaces) allowing for easy transfer into any data manipulation software.

III. Files description

Files contained in the plugin:

1. "Colony_area.java": Java file that contains the code for the plugin and can be edited if changes or additions are desired. This file separates all the wells in the imaged plate and then stacks them for analysis. It also uses the macro "Colony_thresholder.ijm" to select the pixels with cells and remove the background.

2. “Colony_area.class”: Class file that actually executes the code written in the Java file. This is a multiplatform file.
3. “Colony_thresholder.ijm”: Macro file that automatically determines the correct intensity threshold for each well that separates pixels with cells from those with background.
4. “Manual_colony_thresholder.ijm”: Macro that calculates the intensity threshold based on an initial input of the user. This can be used in case the user is not satisfied with the automatic result presented by “Colony_thresholder.ijm”.
5. “Colony_measurer.ijm”: Macro that measures the area percentage covered by cells in each well. It also calculates the intensity percentage based on the area coverage and the intensity at each pixel (See the section “Running the plugin” for details).
6. “ColonyArea User Manual.pdf”: PDF file of this manual describing the plugin and details on how to use it.

They are bundled into a folder "ColonyArea" and freely available for download at http://www.btk.fi/research/research-groups/abankwa/downloads/.

Files saved after running the plugin:

Typically for a file called “***name***.tiff”, the following files are stored in the directory/folder that the user has chosen:

- “wells_***name***.tiff” – image stack of all the wells to be analyzed.
- “thresholded_wells_***name***.tiff” – image stack containing all the thresholded wells.
- “applied_threshold_wells_***name***.txt” – text file containing the thresholds applied to each well.
- “results_thresholded_wells_***name***.txt” – text file containing the final results i.e. well number, area percentage, and intensity percentage.

[Note: You might notice that while the plugin is running there will appear several files in the folder that you have chosen to store your files. These files are only temporarily stored there and are needed for internal work of the plugin. At the end, the plugin will remove them from your folder and permanently delete them.]

Appendix

In this appendix we present you with extra options that have been included in the plugin and that should help you deal with special cases like custom made well plates or when the thresholding is not entirely satisfactory.

A1. Measuring a sub-region of the well:

If you find that after running the plugin on your colony formation assay images, there are wells that included **PARTS** of the background as colonies, you can do an analysis of only a sub-region of the well that avoids the wrongly detected background. Typically, when background is included, this is not sporadic, e.g. it may correspond to: the upper half of the well or the left part of the well or may be a band in the center living you with large areas where cells have been detected correctly and can be analyzed. Follow the next steps to measure only those correctly thresholded areas:

- Use the rectangular selection tool to select the part of well that has been thresholded properly. Since the quantification is done only for the region inside the well, it is not necessary that the selection will lie inside the well (See **Fig. 10**).

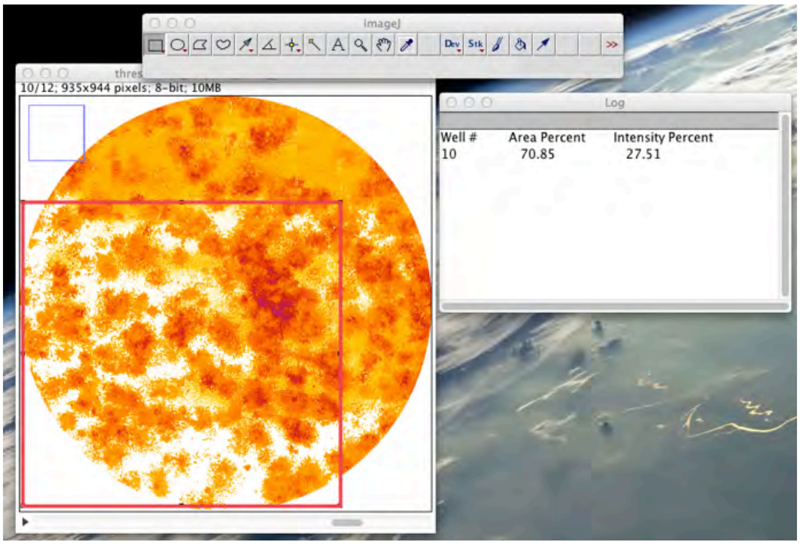

Figure 10: Using a rectangular selection tool to manually choose a sub-region of a well for analysis.

- Then you can go to Plugins→ColonyArea→Colony measurer.
- In the case that you have selected a sub-region of the well, you will be asked to define the range of wells where you want to do this analysis (e.g. all the wells - starting on the first and ending on the last, or only on one or a few of the wells).
- Results will be displayed on a table containing only the wells that you have selected. As both the quantification parameters are ratios (percentage)

therefore the area of the well being measured doesn't have an effect on the results, it only helps to avoid regions where colony detection was not accurate.

[Note: If background has been included with the colonies on the whole well or if cells have been excluded in the whole well, you might want to try the steps described in the section "A3. Incorrect threshold detection" of this appendix.]

A2. Non-standard threshold detection:

When calculating the thresholds that should be applied to each well, this plugin uses the changes on the selected area as a function of the intensity threshold (Area vs. Threshold plot). If for a given well, the intensity in pixels covered by cells is sufficiently different from the intensity of those pixels where there is no cells (background), thresholding can be done with our standard method and those two regions can be easily separated. However, if the well has poor illumination (i.e. shading), images have low resolution or the density of cells is too high or too low, they might be problems separating the cells from the background. In such cases, the plugin will try to use the information from all the other wells of the same image to calculate the correct threshold for that particular well. When this happens, the thresholds window will present a warning asterisk (*). In such cases the user should verify if the applied threshold is correct by comparing the two stacks "wells_***name***.tiff" and "thresholded_wells_***name***.tiff". Just make sure that cells have been detected correctly.

A3. Incorrect threshold detection:

If after comparing the two image stacks named, "wells_***name***.tiff" and "thresholded_wells_***name***.tiff" you find that some of the wells or even all of them have not been thresholded correctly there are 2 possible solutions to this:

1. Improve your image acquisition by having better and more homogenous illumination (i.e. using a scanner instead of a camera, covering the plate completely so that room lights have no effect on scanning) and/or by acquiring your files at a higher resolution (more than 800 dpi)
2. Using the "Manual colony thresholder" tool (see below for details).

A4. Using the "Manual colony thresholder" tool:

In some rare cases, like the case where the plate only consists of wells with extremely high cell density, or extremely low density (null), or a combination of only these extreme cases, the thresholds detected may be actually very low or very high compared to the correct values. This leads to a gross error in the detection of colonies. In this case, the user should use the option of "Manual colony thresholder" following the next steps:

- Open the cropped wells stack saved before while running the "ColonyArea" plugin or just select the stack of cropped wells generated after running "Colony area". File "wells_name.tiff".

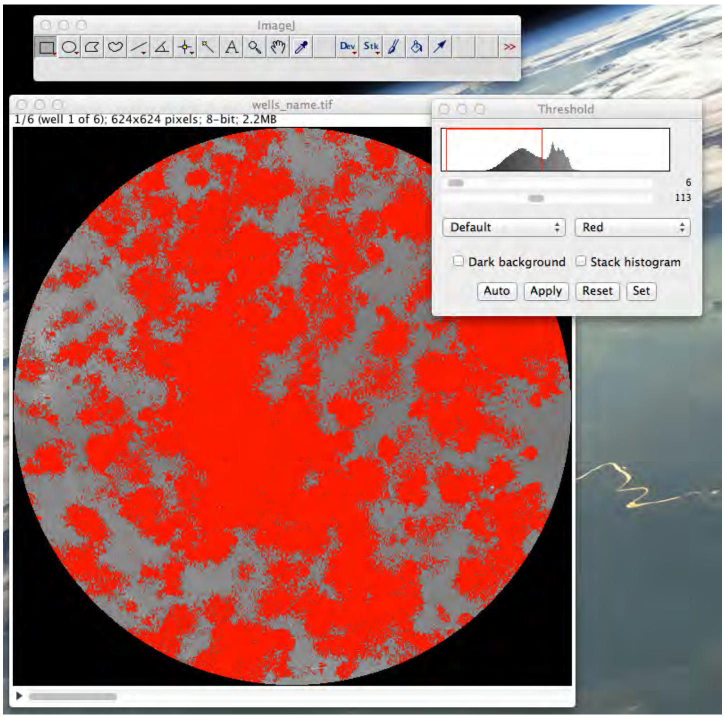

Figure 11: Determining the reference threshold of a reference well in case of failure of automatic thresholding.

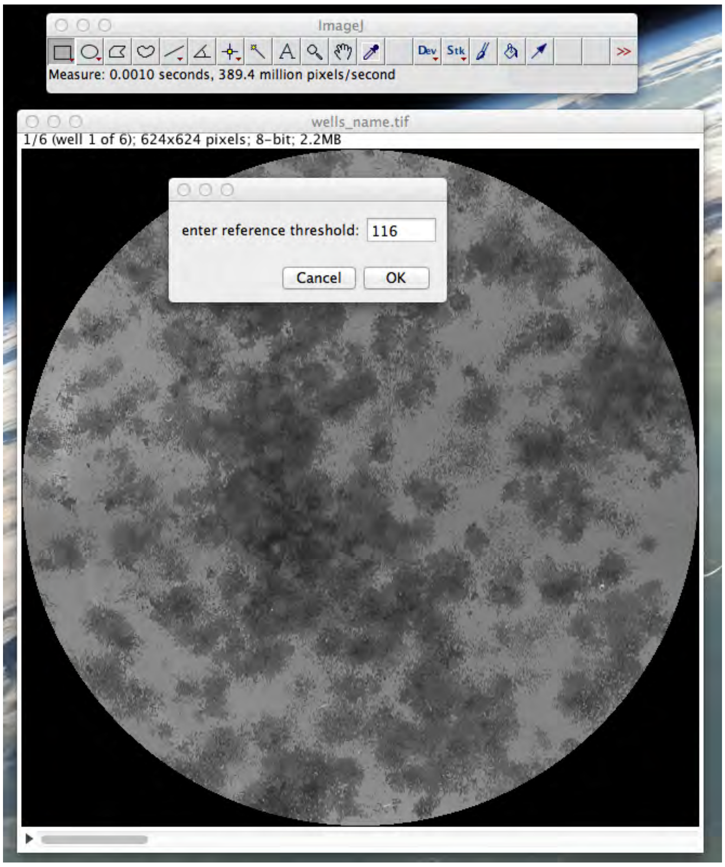

Figure 12: Running the "Manual colony thresholder" tool: setting up the reference well and the reference threshold in that well.

- Select a well of your choice as your reference well. Go to Image→Adjust→Threshold
- Slide the lower threshold slider to 1.
- Slide the upper threshold slider such that all or most of the colonies are covered with red while almost zero background is covered with it.
- Take note of this reference threshold and close the threshold window without applying the threshold (See **Fig. 11**).
- Go to Plugins→ColonyArea→Manual colony thresholder
- Enter your reference well number and your reference threshold. The dialog will have a reference threshold already detected, you may choose to ignore it or use it (See **Fig. 12**).
- The plugin will threshold these wells and you would again have two stacks, one of the original wells stack and other of the thresholded wells.
- Compare them and see if results are satisfactory this time. If so, proceed to quantify using the “Colony measurer” tool as already described in this manual.

A5. Incorrect cropping:

For this plugin to perform correctly, it requires that the selection/cropping of the ROI will be done precisely enough so that the wells can be identified and separated. The position of the wells in the image is determined by the type of plate (6, 12, 24-well or custom well plate); by the number of wells selected (number of wells in a row and in a column); and by the accuracy of the cropping. If the plugin determines that you have not cropped the image to the required degree of accuracy (meaning, the proportions between width and height are not as expected), it will tell you that cropping is incorrect. Based on the expected proportion for your input parameters, the plugin will tell you the maximum error in cropping, and also the direction (horizontal/vertical). This is done to provide a rough estimate of where the user went wrong in cropping. The error displayed is the value in pixels that has been cropped less than required, assuming that the cropping in the other direction was done satisfactorily. (e.g. If the approximate max error in cropping is of the order of 150 pixels horizontally, this will mean that, if the cropping in vertical direction is accurate, then you have to crop 150 pixels more horizontally).

A6. Using a custom plate:

If there is a case when you have performed your experiment not in a standard 6, 12 or 24-well plate but instead on a custom plate, you can still do the analysis by selecting the option 4 “Custom plate” in the step 4 of the section “II: Thresholding and colony quantification using Colony_area”, of this manual. In such case you will be asked to enter 3 parameters about your custom plate (See **Fig. 13** for details):

- $K2 = D2/D1$, the ratio between the thickness of the well’s wall and the internal diameter of the well.
- $K3 = D3/D1$, the ratio of the well spacing (shortest distance between the outer walls of two adjacent wells) to that of internal diameter of the well.

- N, percentage of diameter reduction (set as 5% by default). The actual area of the well that is processed consists of an area smaller than that of the actual well. This is primarily due to two reasons:
  a) To prevent that the wall of the well is included in the area to be processed. This could happen if the image is not straightened or the selection of region of interest is not done to a required degree of accuracy.
  b) The region near the wall shows unpredictable behavior in colony formation and that is why this area is neglected from the analysis. Reduction of the default 5% corresponds to about 2 mm in the case of a 12 well plate.

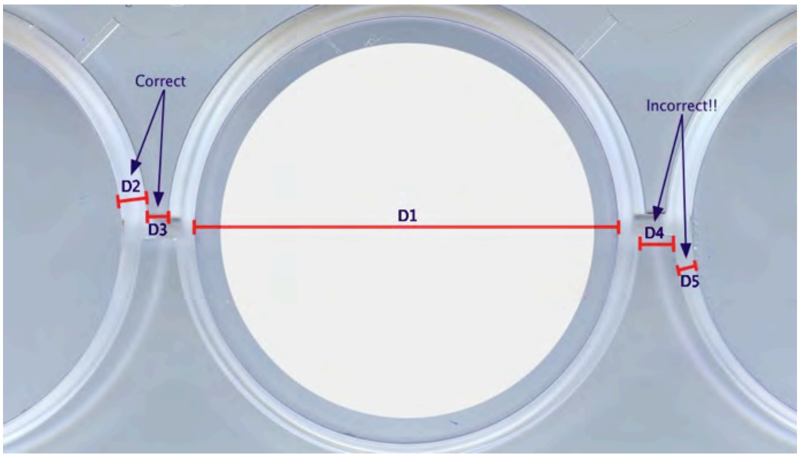

Figure 13: Parameters calculation for a customized plate. $D2$and $D3$ are the correct way of measuring the well's wall thickness (maximum thickness) and the inter-well distance (minimum distance) while$D4$ and $D5$ are the incorrect way of measuring them. The final area to be analyzed for colonies (white area) corresponds to the area of the well reducing its diameter $D1$ by N%.

Steps involved in the processing

1. Colony_area.java:
    a. Conversion to 8 bit gray scale
    b. Formation of template
    c. Formation of image stack of all the wells
    d. Saving the template and the image stack
    e. Calling <Colony_thresholder.ijm>

2. Colony_thresholder.ijm
    a. The maximum intensity in the wells is computed and all the wells are scaled in such a way so that their maximum intensity is 200. (This is done to counter the effect of non-uniform illumination, and also because of the fact that the scanner may have applied backlight correction, or it may not have. Thus illumination is corrected. 200 is an abstract value to allow a better visualization for the user, and it has no effect on the results.)
    b. Acquisition well per well of an array of area detected with increasing threshold values.
    c. 5 thresholds are computed from this array, and the best one is stored as the threshold.
    d. If no threshold is detected or if the threshold is too low compared to other cells, threshold is recorded as 0 for that well. (For comparison, an average is computed with the values that are fit to be set as a threshold.)
    e. For images with threshold set as 0 because of non-detection/ wrong detection, a new threshold is scaled back from the average of thresholds of the scaled images.
    f. A binary mask is calculated from the image stack containing 255 at pixels which are detected as colonies and 0 at pixels detected as background. This mask is multiplied with the scaled, inverted image of the well to get an image, which is varying in pixel density according to the original image where, the colonies are detected, and is 0 where background is detected. (Images were inverted as the gray scale version of the image had the cells as darker parts, and the background as the lighter part)

3. Colony_measurer.ijm
    a. Once it is called, it checks for a selection.
    b. If it encounters a selection, it computes a ratio that tells us how much of that part of that selection is a part of our well and must be analyzed. In the case of a selection it also asks the user to select the user to select the well limits (from which well to which well).If no selection is detected then it proceeds to measure all wells.
    c. It measures the percentage of area covered by colonies in the well.

$$colony\ area\ \% = \frac{\#\ of\ pixels\ in\ the\ region\ with\ an\ intensity\ above\ zero}{Total\ \#\ of\ pixels\ in\ the\ same\ region}\ x\ 100$$

    d. It also measures well-wise the intensity percentage, which is defined as: Intensity_percentage=sum of intensities in the well or the selected region of well*100 divided by the maximum sum of intensities possible for that area

$$colony\ intensity\ \% = \frac{\sum pixel\ intensities\ in\ a\ region}{\sum maximum\ intensities\ possible\ in\ the\ same\ region} x\ 100$$

[Note: sum of maximum intensities can be rewritten as 255*area of well or region.]
