# Supplementary material for: ColonyArea: An ImageJ Plugin to Automatically Quantify Colony Formation in Clonogenic Assays
Source: PLoS One. 2014 Mar 19;9(3):e92444. doi: 10.1371/journal.pone.0092444 (PMC3960247; doi:10.1371/journal.pone.0092444)
Supplement: Information S1 — The additional file ‘ColonyArea.zip’ contains the plugin installation files and user manual. In this zip-compressed file, we provide seven plugin files: Colony_area.java; Colony_area.class; Colony_thresholder.ijm; Manual_colony_thresholder.ijm; Colony_measurer.ijm; a user manual file, ColonyArea_manual.pdf and; the GNU general public license, gpl.rtf. (ZIP) [file pone.0092444.s005.zip › ColonyArea/gpl.rtf]

                    GNU GENERAL PUBLIC LICENSE                       Version 3, 29 June 2007 Copyright (C) 2007 Free Software Foundation, Inc. <http://fsf.org/> Everyone is permitted to copy and distribute verbatim copies of this license document, but changing it is not allowed.                            Preamble  The GNU General Public License is a free, copyleft license forsoftware and other kinds of works.  The licenses for most software and other practical works are designedto take away your freedom to share and change the works.  By contrast,the GNU General Public License is intended to guarantee your freedom toshare and change all versions of a program--to make sure it remains freesoftware for all its users.  We, the Free Software Foundation, use theGNU General Public License for most of our software; it applies also toany other work released this way by its authors.  You can apply it toyour programs, too.  When we speak of free software, we are referring to freedom, notprice.  Our General Public Licenses are designed to make sure that youhave the freedom to distribute copies of free software (and charge forthem if you wish), that you receive source code or can get it if youwant it, that you can change the software or use pieces of it in newfree programs, and that you know you can do these things.  To protect your rights, we need to prevent others from denying youthese rights or asking you to surrender the rights.  Therefore, you havecertain responsibilities if you distribute copies of the software, or ifyou modify it: responsibilities to respect the freedom of others.  For example, if you distribute copies of such a program, whethergratis or for a fee, you must pass on to the recipients the samefreedoms that you received.  You must make sure that they, too, receiveor can get the source code.  And you must show them these terms so theyknow their rights.  Developers that use the GNU GPL protect your rights with two steps:(1) assert copyright on the software, and (2) offer you this Licensegiving you legal permission to copy, distribute and/or modify it.  For the developers' and authors' protection, the GPL clearly explainsthat there is no warranty for this free software.  For both users' andauthors' sake, the GPL requires that modified versions be marked aschanged, so that their problems will not be attributed erroneously toauthors of previous versions.  Some devices are designed to deny users access to install or runmodified versions of the software inside them, although the manufacturercan do so.  This is fundamentally incompatible with the aim ofprotecting users' freedom to change the software.  The systematicpattern of such abuse occurs in the area of products for individuals touse, which is precisely where it is most unacceptable.  Therefore, wehave designed this version of the GPL to prohibit the practice for thoseproducts.  If such problems arise substantially in other domains, westand ready to extend this provision to those domains in future versionsof the GPL, as needed to protect the freedom of users.  Finally, every program is threatened constantly by software patents.States should not allow patents to restrict development and use ofsoftware on general-purpose computers, but in those that do, we wish toavoid the special danger that patents applied to a free program couldmake it effectively proprietary.  To prevent this, the GPL assures thatpatents cannot be used to render the program non-free.  The precise terms and conditions for copying, distribution andmodification follow.                       TERMS AND CONDITIONS  0. Definitions.  "This License" refers to version 3 of the GNU General Public License.  "Copyright" also means copyright-like laws that apply to other kinds ofworks, such as semiconductor masks.  "The Program" refers to any copyrightable work licensed under thisLicense.  Each licensee is addressed as "you".  "Licensees" and"recipients" may be individuals or organizations.  To "modify" a work means to copy from or adapt all or part of the workin a fashion requiring copyright permission, other than the making of anexact copy.  The resulting work is called a "modified version" of theearlier work or a work "based on" the earlier work.  A "covered work" means either the unmodified Program or a work basedon the Program.  To "propagate" a work means to do anything with it that, withoutpermission, would make you directly or secondarily liable forinfringement under applicable copyright law, except executing it on acomputer or modifying a private copy.  Propagation includes copying,distribution (with or without modification), making available to thepublic, and in some countries other activities as well.  To "convey" a work means any kind of propagation that enables otherparties to make or receive copies.  Mere interaction with a user througha computer network, with no transfer of a copy, is not conveying.  An interactive user interface displays "Appropriate Legal Notices"to the extent that it includes a convenient and prominently visiblefeature that (1) displays an appropriate copyright notice, and (2)tells the user that there is no warranty for the work (except to theextent that warranties are provided), that licensees may convey thework under this License, and how to view a copy of this License.  Ifthe interface presents a list of user commands or options, such as amenu, a prominent item in the list meets this criterion.  1. Source Code.  The "source code" for a work means the preferred form of the workfor making modifications to it.  "Object code" means any non-sourceform of a work.  A "Standard Interface" means an interface that either is an officialstandard defined by a recognized standards body, or, in the case ofinterfaces specified for a particular programming language, one thatis widely used among developers working in that language.  The "System Libraries" of an executable work include anything, otherthan the work as a whole, that (a) is included in the normal form ofpackaging a Major Component, but which is not part of that MajorComponent, and (b) serves only to enable use of the work with thatMajor Component, or to implement a Standard Interface for which animplementation is available to the public in source code form.  A"Major Component", in this context, means a major essential component(kernel, window system, and so on) of the specific operating system(if any) on which the executable work runs, or a compiler used toproduce the work, or an object code interpreter used to run it.  The "Corresponding Source" for a work in object code form means allthe source code needed to generate, install, and (for an executablework) run the object code and to modify the work, including scripts tocontrol those activities.  However, it does not include the work'sSystem Libraries, or general-purpose tools or generally available freeprograms which are used unmodified in performing those activities butwhich are not part of the work.  For example, Corresponding Sourceincludes interface definition files associated with source files forthe work, and the source code for shared libraries and dynamicallylinked subprograms that the work is specifically designed to require,such as by intimate data communication or control flow between thosesubprograms and other parts of the work.  The Corresponding Source need not include anything that userscan regenerate automatically from other parts of the CorrespondingSource.  The Corresponding Source for a work in source code form is thatsame work.  2. Basic Permissions.  All rights granted under this License are granted for the term ofcopyright on the Program, and are irrevocable provided the statedconditions are met.  This License explicitly affirms your unlimitedpermission to run the unmodified Program.  The output from running acovered work is covered by this License only if the output, given itscontent, constitutes a covered work.  This License acknowledges yourrights of fair use or other equivalent, as provided by copyright law.  You may make, run and propagate covered works that you do notconvey, without conditions so long as your license otherwise remainsin force.  You may convey covered works to others for the sole purposeof having them make modifications exclusively for you, or provide youwith facilities for running those works, provided that you comply withthe terms of this License in conveying all material for which you donot control copyright.  Those thus making or running the covered worksfor you must do so exclusively on your behalf, under your directionand control, on terms that prohibit them from making any copies ofyour copyrighted material outside their relationship with you.  Conveying under any other circumstances is permitted solely underthe conditions stated below.  Sublicensing is not allowed; section 10makes it unnecessary.  3. Protecting Users' Legal Rights From Anti-Circumvention Law.  No covered work shall be deemed part of an effective technologicalmeasure under any applicable law fulfilling obligations under article11 of the WIPO copyright treaty adopted on 20 December 1996, orsimilar laws prohibiting or restricting circumvention of suchmeasures.  When you convey a covered work, you waive any legal power to forbidcircumvention of technological measures to the extent such circumventionis effected by exercising rights under this License with respect tothe covered work, and you disclaim any intention to limit operation ormodification of the work as a means of enforcing, against the work'susers, your or third parties' legal rights to forbid circumvention oftechnological measures.  4. Conveying Verbatim Copies.  You may convey verbatim copies of the Program's source code as youreceive it, in any medium, provided that you conspicuously andappropriately publish on each copy an appropriate copyright notice;keep intact all notices stating that this License and anynon-permissive terms added in accord with section 7 apply to the code;keep intact all notices of the absence of any warranty; and give allrecipients a copy of this License along with the Program.  You may charge any price or no price for each copy that you convey,and you may offer support or warranty protection for a fee.  5. Conveying Modified Source Versions.  You may convey a work based on the Program, or the modifications toproduce it from the Program, in the form of source code under theterms of section 4, provided that you also meet all of these conditions:    a) The work must carry prominent notices stating that you modified    it, and giving a relevant date.    b) The work must carry prominent notices stating that it is    released under this License and any conditions added under section    7.  This requirement modifies the requirement in section 4 to    "keep intact all notices".    c) You must license the entire work, as a whole, under this    License to anyone who comes into possession of a copy.  This    License will therefore apply, along with any applicable section 7    additional terms, to the whole of the work, and all its parts,    regardless of how they are packaged.  This License gives no    permission to license the work in any other way, but it does not    invalidate such permission if you have separately received it.    d) If the work has interactive user interfaces, each must display    Appropriate Legal Notices; however, if the Program has interactive    interfaces that do not display Appropriate Legal Notices, your    work need not make them do so.  A compilation of a covered work with other separate and independentworks, which are not by their nature extensions of the covered work,and which are not combined with it such as to form a larger program,in or on a volume of a storage or distribution medium, is called an"aggregate" if the compilation and its resulting copyright are notused to limit the access or legal rights of the compilation's usersbeyond what the individual works permit.  Inclusion of a covered workin an aggregate does not cause this License to apply to the otherparts of the aggregate.  6. Conveying Non-Source Forms.  You may convey a covered work in object code form under the termsof sections 4 and 5, provided that you also convey themachine-readable Corresponding Source under the terms of this License,in one of these ways:    a) Convey the object code in, or embodied in, a physical product    (including a physical distribution medium), accompanied by the    Corresponding Source fixed on a durable physical medium    customarily used for software interchange.    b) Convey the object code in, or embodied in, a physical product    (including a physical distribution medium), accompanied by a    written offer, valid for at least three years and valid for as    long as you offer spare parts or customer support for that product    model, to give anyone who possesses the object code either (1) a    copy of the Corresponding Source for all the software in the    product that is covered by this License, on a durable physical    medium customarily used for software interchange, for a price no    more than your reasonable cost of physically performing this    conveying of source, or (2) access to copy the    Corresponding Source from a network server at no charge.    c) Convey individual copies of the object code with a copy of the    written offer to provide the Corresponding Source.  This    alternative is allowed only occasionally and noncommercially, and    only if you received the object code with such an offer, in accord    with subsection 6b.    d) Convey the object code by offering access from a designated    place (gratis or for a charge), and offer equivalent access to the    Corresponding Source in the same way through the same place at no    further charge.  You need not require recipients to copy the    Corresponding Source along with the object code.  If the place to    copy the object code is a network server, the Corresponding Source    may be on a different server (operated by you or a third party)    that supports equivalent copying facilities, provided you maintain    clear directions next to the object code saying where to find the    Corresponding Source.  Regardless of what server hosts the    Corresponding Source, you remain obligated to ensure that it is    available for as long as needed to satisfy these requirements.    e) Convey the object code using peer-to-peer transmission, provided    you inform other peers where the object code and Corresponding    Source of the work are being offered to the general public at no    charge under subsection 6d.  A separable portion of the object code, whose source code is excludedfrom the Corresponding Source as a System Library, need not beincluded in conveying the object code work.  A "User Product" is either (1) a "consumer product", which means anytangible personal property which is normally used for personal, family,or household purposes, or (2) anything designed or sold for incorporationinto a dwelling.  In determining whether a product is a consumer product,doubtful cases shall be resolved in favor of coverage.  For a particularproduct received by a particular user, "normally used" refers to atypical or common use of that class of product, regardless of the statusof the particular user or of the way in which the particular useractually uses, or expects or is expected to use, the product.  A productis a consumer product regardless of whether the product has substantialcommercial, industrial or non-consumer uses, unless such uses representthe only significant mode of use of the product.  "Installation Information" for a User Product means any methods,procedures, authorization keys, or other information required to installand execute modified versions of a covered work in that User Product froma modified version of its Corresponding Source.  The information mustsuffice to ensure that the continued functioning of the modified objectcode is in no case prevented or interfered with solely becausemodification has been made.  If you convey an object code work under this section in, or with, orspecifically for use in, a User Product, and the conveying occurs aspart of a transaction in which the right of possession and use of theUser Product is transferred to the recipient in perpetuity or for afixed term (regardless of how the transaction is characterized), theCorresponding Source conveyed under this section must be accompaniedby the Installation Information.  But this requirement does not applyif neither you nor any third party retains the ability to installmodified object code on the User Product (for example, the work hasbeen installed in ROM).  The requirement to provide Installation Information does not include arequirement to continue to provide support service, warranty, or updatesfor a work that has been modified or installed by the recipient, or forthe User Product in which it has been modified or installed.  Access to anetwork may be denied when the modification itself materially andadversely affects the operation of the network or violates the rules andprotocols for communication across the network.  Corresponding Source conveyed, and Installation Information provided,in accord with this section must be in a format that is publiclydocumented (and with an implementation available to the public insource code form), and must require no special password or key forunpacking, reading or copying.  7. Additional Terms.  "Additional permissions" are terms that supplement the terms of thisLicense by making exceptions from one or more of its conditions.Additional permissions that are applicable to the entire Program shallbe treated as though they were included in this License, to the extentthat they are valid under applicable law.  If additional permissionsapply only to part of the Program, that part may be used separatelyunder those permissions, but the entire Program remains governed bythis License without regard to the additional permissions.  When you convey a copy of a covered work, you may at your optionremove any additional permissions from that copy, or from any part ofit.  (Additional permissions may be written to require their ownremoval in certain cases when you modify the work.)  You may placeadditional permissions on material, added by you to a covered work,for which you have or can give appropriate copyright permission.  Notwithstanding any other provision of this License, for material youadd to a covered work, you may (if authorized by the copyright holders ofthat material) supplement the terms of this License with terms:    a) Disclaiming warranty or limiting liability differently from the    terms of sections 15 and 16 of this License; or    b) Requiring preservation of specified reasonable legal notices or    author attributions in that material or in the Appropriate Legal    Notices displayed by works containing it; or    c) Prohibiting misrepresentation of the origin of that material, or    requiring that modified versions of such material be marked in    reasonable ways as different from the original version; or    d) Limiting the use for publicity purposes of names of licensors or    authors of the material; or    e) Declining to grant rights under trademark law for use of some    trade names, trademarks, or service marks; or    f) Requiring indemnification of licensors and authors of that    material by anyone who conveys the material (or modified versions of    it) with contractual assumptions of liability to the recipient, for    any liability that these contractual assumptions directly impose on    those licensors and authors.  All other non-permissive additional terms are considered "furtherrestrictions" within the meaning of section 10.  If the Program as youreceived it, or any part of it, contains a notice stating that it isgoverned by this License along with a term that is a furtherrestriction, you may remove that term.  If a license document containsa further restriction but permits relicensing or conveying under thisLicense, you may add to a covered work material governed by the termsof that license document, provided that the further restriction doesnot survive such relicensing or conveying.  If you add terms to a covered work in accord with this section, youmust place, in the relevant source files, a statement of theadditional terms that apply to those files, or a notice indicatingwhere to find the applicable terms.  Additional terms, permissive or non-permissive, may be stated in theform of a separately written license, or stated as exceptions;the above requirements apply either way.  8. Termination.  You may not propagate or modify a covered work except as expresslyprovided under this License.  Any attempt otherwise to propagate ormodify it is void, and will automatically terminate your rights underthis License (including any patent licenses granted under the thirdparagraph of section 11).  However, if you cease all violation of this License, then yourlicense from a particular copyright holder is reinstated (a)provisionally, unless and until the copyright holder explicitly andfinally terminates your license, and (b) permanently, if the copyrightholder fails to notify you of the violation by some reasonable meansprior to 60 days after the cessation.  Moreover, your license from a particular copyright holder isreinstated permanently if the copyright holder notifies you of theviolation by some reasonable means, this is the first time you havereceived notice of violation of this License (for any work) from thatcopyright holder, and you cure the violation prior to 30 days afteryour receipt of the notice.  Termination of your rights under this section does not terminate thelicenses of parties who have received copies or rights from you underthis License.  If your rights have been terminated and not permanentlyreinstated, you do not qualify to receive new licenses for the samematerial under section 10.  9. Acceptance Not Required for Having Copies.  You are not required to accept this License in order to receive orrun a copy of the Program.  Ancillary propagation of a covered workoccurring solely as a consequence of using peer-to-peer transmissionto receive a copy likewise does not require acceptance.  However,nothing other than this License grants you permission to propagate ormodify any covered work.  These actions infringe copyright if you donot accept this License.  Therefore, by modifying or propagating acovered work, you indicate your acceptance of this License to do so.  10. Automatic Licensing of Downstream Recipients.  Each time you convey a covered work, the recipient automaticallyreceives a license from the original licensors, to run, modify andpropagate that work, subject to this License.  You are not responsiblefor enforcing compliance by third parties with this License.  An "entity transaction" is a transaction transferring control of anorganization, or substantially all assets of one, or subdividing anorganization, or merging organizations.  If propagation of a coveredwork results from an entity transaction, each party to thattransaction who receives a copy of the work also receives whateverlicenses to the work the party's predecessor in interest had or couldgive under the previous paragraph, plus a right to possession of theCorresponding Source of the work from the predecessor in interest, ifthe predecessor has it or can get it with reasonable efforts.  You may not impose any further restrictions on the exercise of therights granted or affirmed under this License.  For example, you maynot impose a license fee, royalty, or other charge for exercise ofrights granted under this License, and you may not initiate litigation(including a cross-claim or counterclaim in a lawsuit) alleging thatany patent claim is infringed by making, using, selling, offering forsale, or importing the Program or any portion of it.  11. Patents.  A "contributor" is a copyright holder who authorizes use under thisLicense of the Program or a work on which the Program is based.  Thework thus licensed is called the contributor's "contributor version".  A contributor's "essential patent claims" are all patent claimsowned or controlled by the contributor, whether already acquired orhereafter acquired, that would be infringed by some manner, permittedby this License, of making, using, or selling its contributor version,but do not include claims that would be infringed only as aconsequence of further modification of the contributor version.  Forpurposes of this definition, "control" includes the right to grantpatent sublicenses in a manner consistent with the requirements ofthis License.  Each contributor grants you a non-exclusive, worldwide, royalty-freepatent license under the contributor's essential patent claims, tomake, use, sell, offer for sale, import and otherwise run, modify andpropagate the contents of its contributor version.  In the following three paragraphs, a "patent license" is any expressagreement or commitment, however denominated, not to enforce a patent(such as an express permission to practice a patent or covenant not tosue for patent infringement).  To "grant" such a patent license to aparty means to make such an agreement or commitment not to enforce apatent against the party.  If you convey a covered work, knowingly relying on a patent license,and the Corresponding Source of the work is not available for anyoneto copy, free of charge and under the terms of this License, through apublicly available network server or other readily accessible means,then you must either (1) cause the Corresponding Source to be soavailable, or (2) arrange to deprive yourself of the benefit of thepatent license for this particular work, or (3) arrange, in a mannerconsistent with the requirements of this License, to extend the patentlicense to downstream recipients.  "Knowingly relying" means you haveactual knowledge that, but for the patent license, your conveying thecovered work in a country, or your recipient's use of the covered workin a country, would infringe one or more identifiable patents in thatcountry that you have reason to believe are valid.  If, pursuant to or in connection with a single transaction orarrangement, you convey, or propagate by procuring conveyance of, acovered work, and grant a patent license to some of the partiesreceiving the covered work authorizing them to use, propagate, modifyor convey a specific copy of the covered work, then the patent licenseyou grant is automatically extended to all recipients of the coveredwork and works based on it.  A patent license is "discriminatory" if it does not include withinthe scope of its coverage, prohibits the exercise of, or isconditioned on the non-exercise of one or more of the rights that arespecifically granted under this License.  You may not convey a coveredwork if you are a party to an arrangement with a third party that isin the business of distributing software, under which you make paymentto the third party based on the extent of your activity of conveyingthe work, and under which the third party grants, to any of theparties who would receive the covered work from you, a discriminatorypatent license (a) in connection with copies of the covered workconveyed by you (or copies made from those copies), or (b) primarilyfor and in connection with specific products or compilations thatcontain the covered work, unless you entered into that arrangement,or that patent license was granted, prior to 28 March 2007.  Nothing in this License shall be construed as excluding or limitingany implied license or other defenses to infringement that mayotherwise be available to you under applicable patent law.  12. No Surrender of Others' Freedom.  If conditions are imposed on you (whether by court order, agreement orotherwise) that contradict the conditions of this License, they do notexcuse you from the conditions of this License.  If you cannot convey acovered work so as to satisfy simultaneously your obligations under thisLicense and any other pertinent obligations, then as a consequence you maynot convey it at all.  For example, if you agree to terms that obligate youto collect a royalty for further conveying from those to whom you conveythe Program, the only way you could satisfy both those terms and thisLicense would be to refrain entirely from conveying the Program.  13. Use with the GNU Affero General Public License.  Notwithstanding any other provision of this License, you havepermission to link or combine any covered work with a work licensedunder version 3 of the GNU Affero General Public License into a singlecombined work, and to convey the resulting work.  The terms of thisLicense will continue to apply to the part which is the covered work,but the special requirements of the GNU Affero General Public License,section 13, concerning interaction through a network will apply to thecombination as such.  14. Revised Versions of this License.  The Free Software Foundation may publish revised and/or new versions ofthe GNU General Public License from time to time.  Such new versions willbe similar in spirit to the present version, but may differ in detail toaddress new problems or concerns.  Each version is given a distinguishing version number.  If theProgram specifies that a certain numbered version of the GNU GeneralPublic License "or any later version" applies to it, you have theoption of following the terms and conditions either of that numberedversion or of any later version published by the Free SoftwareFoundation.  If the Program does not specify a version number of theGNU General Public License, you may choose any version ever publishedby the Free Software Foundation.  If the Program specifies that a proxy can decide which futureversions of the GNU General Public License can be used, that proxy'spublic statement of acceptance of a version permanently authorizes youto choose that version for the Program.  Later license versions may give you additional or differentpermissions.  However, no additional obligations are imposed on anyauthor or copyright holder as a result of your choosing to follow alater version.  15. Disclaimer of Warranty.  THERE IS NO WARRANTY FOR THE PROGRAM, TO THE EXTENT PERMITTED BYAPPLICABLE LAW.  EXCEPT WHEN OTHERWISE STATED IN WRITING THE COPYRIGHTHOLDERS AND/OR OTHER PARTIES PROVIDE THE PROGRAM "AS IS" WITHOUT WARRANTYOF ANY KIND, EITHER EXPRESSED OR IMPLIED, INCLUDING, BUT NOT LIMITED TO,THE IMPLIED WARRANTIES OF MERCHANTABILITY AND FITNESS FOR A PARTICULARPURPOSE.  THE ENTIRE RISK AS TO THE QUALITY AND PERFORMANCE OF THE PROGRAMIS WITH YOU.  SHOULD THE PROGRAM PROVE DEFECTIVE, YOU ASSUME THE COST OFALL NECESSARY SERVICING, REPAIR OR CORRECTION.  16. Limitation of Liability.  IN NO EVENT UNLESS REQUIRED BY APPLICABLE LAW OR AGREED TO IN WRITINGWILL ANY COPYRIGHT HOLDER, OR ANY OTHER PARTY WHO MODIFIES AND/OR CONVEYSTHE PROGRAM AS PERMITTED ABOVE, BE LIABLE TO YOU FOR DAMAGES, INCLUDING ANYGENERAL, SPECIAL, INCIDENTAL OR CONSEQUENTIAL DAMAGES ARISING OUT OF THEUSE OR INABILITY TO USE THE PROGRAM (INCLUDING BUT NOT LIMITED TO LOSS OFDATA OR DATA BEING RENDERED INACCURATE OR LOSSES SUSTAINED BY YOU OR THIRDPARTIES OR A FAILURE OF THE PROGRAM TO OPERATE WITH ANY OTHER PROGRAMS),EVEN IF SUCH HOLDER OR OTHER PARTY HAS BEEN ADVISED OF THE POSSIBILITY OFSUCH DAMAGES.  17. Interpretation of Sections 15 and 16.  If the disclaimer of warranty and limitation of liability providedabove cannot be given local legal effect according to their terms,reviewing courts shall apply local law that most closely approximatesan absolute waiver of all civil liability in connection with theProgram, unless a warranty or assumption of liability accompanies acopy of the Program in return for a fee.                     END OF TERMS AND CONDITIONS            How to Apply These Terms to Your New Programs  If you develop a new program, and you want it to be of the greatestpossible use to the public, the best way to achieve this is to make itfree software which everyone can redistribute and change under these terms.  To do so, attach the following notices to the program.  It is safestto attach them to the start of each source file to most effectivelystate the exclusion of warranty; and each file should have at leastthe "copyright" line and a pointer to where the full notice is found.    <one line to give the program's name and a brief idea of what it does.>    Copyright (C) <year>  <name of author>    This program is free software: you can redistribute it and/or modify    it under the terms of the GNU General Public License as published by    the Free Software Foundation, either version 3 of the License, or    (at your option) any later version.    This program is distributed in the hope that it will be useful,    but WITHOUT ANY WARRANTY; without even the implied warranty of    MERCHANTABILITY or FITNESS FOR A PARTICULAR PURPOSE.  See the    GNU General Public License for more details.    You should have received a copy of the GNU General Public License    along with this program.  If not, see <http://www.gnu.org/licenses/>.Also add information on how to contact you by electronic and paper mail.  If the program does terminal interaction, make it output a shortnotice like this when it starts in an interactive mode:    <program>  Copyright (C) <year>  <name of author>    This program comes with ABSOLUTELY NO WARRANTY; for details type `show w'.    This is free software, and you are welcome to redistribute it    under certain conditions; type `show c' for details.The hypothetical commands `show w' and `show c' should show the appropriateparts of the General Public License.  Of course, your program's commandsmight be different; for a GUI interface, you would use an "about box".  You should also get your employer (if you work as a programmer) or school,if any, to sign a "copyright disclaimer" for the program, if necessary.For more information on this, and how to apply and follow the GNU GPL, see<http://www.gnu.org/licenses/>.  The GNU General Public License does not permit incorporating your programinto proprietary programs.  If your program is a subroutine library, youmay consider it more useful to permit linking proprietary applications withthe library.  If this is what you want to do, use the GNU Lesser GeneralPublic License instead of this License.  But first, please read<http://www.gnu.org/philosophy/why-not-lgpl.html>.
